# SDC2 and FN as cargo proteins in circulating extracellular vesicles in obese breast cancer patients with lymph node metastasis

**DOI:** 10.1038/s41598-025-17638-2

**Published:** 2025-09-12

**Authors:** Liali Yousef Talat, Ghada Mohamed, Maher H. Ibraheem, Amr Ahmed WalyEldeen, Hebatallah Hassan, Sherif Abdelaziz Ibrahim

**Affiliations:** 1 Department of Zoology, Faculty of Science, Cairo University, Giza, 12613 Egypt; 2 Department of Pathology, National Cancer Institute, Cairo University, Cairo, 11796 Egypt; 3 Department of Surgical Oncology, National Cancer Institute, Cairo University, Cairo, 11796 Egypt; 4 Department of Surgical Oncology, Baheya Centre for Early Detection and Treatment of Breast Cancer, Giza, Egypt

**Keywords:** Extracellular vesicles, Microvesicles, Breast cancer, Metastasis, SDC2, FN, Cancer, Breast cancer

## Abstract

Lymph node metastasis (LNM) is a pivotal determinant of breast cancer (BC) patient prognosis and treatment efficacy. Cell surface heparan sulfate proteoglycans (HSPGs), namely, syndecan-1 (SDC1), SDC2, and SDC4, are involved in cancer progression, metastasis, and regulate extracellular vesicles (EVs) biogenesis, including the microvesicles (MVs). This study analyzed MV-enriched EVs isolated from blood plasma of BC patients with negative (*n* = 19) and positive (*n* = 20) LNM (nLNM and pLNM, respectively) using differential centrifugation. Western blot analysis revealed significantly elevated SDC2 levels in MV-enriched EVs from pLNM cases compared to nLNM. Additionally, fibronectin (FN), a SDC2-interacting protein identified through STRING analysis, was also upregulated in pLNM MV-enriched EVs. In contrast, qRT-PCR showed reduced *SDC2* (*P* < 0.01) and *FN* (*P* < 0.05) mRNA levels in tumor tissues of pLNM patients compared to nLNM. ROC analysis highlighted the diagnostic value of *SDC2* (AUC: 0.8376) and *FN* (AUC: 0.8803) mRNA in differentiating LNM status. Bioinformatics analyses further confirmed the association of SDC2 and FN expression with BC staging and prognosis. These findings underscore the potential of circulating MV-enriched EV-associated SDC2 and FN, along with their tumor tissue mRNA expression, as potential predictive biomarkers for LNM and chemotherapy response in chemotherapy-naïve obese BC patients.

**Supplementary Information:**

The online version contains supplementary material available at 10.1038/s41598-025-17638-2.

## Introduction

In 2024, breast cancer is projected to be the most frequently diagnosed cancer worldwide and the second leading cause of cancer-related mortality among women^1^. By 2040, the annual incidence of breast cancer is predicted to exceed 3 million new cases, with an estimated 1 million associated deaths globally, due to the combined effects of population aging and expansion^2^. In Egypt, breast cancer accounts for approximately 38.8% of all female malignant tumors^3^.

Obesity has been found to markedly raise the risk of breast cancer development in women, regardless of their menopausal status and negatively affect recurrence rates and overall survival (OS)^4,^ as breast cancer patients with obesity exhibit a 30% higher mortality than those with a normal body mass index (BMI)^5^. It has been found that obesity was correlated with the presence of enlarged tumors, the development of lymph node metastasis (LNM), poorer distant disease-free and OS^6^. In addition, obesity has an impact on multiple aspects of breast cancer, such as interruption of the immune system, changes in the extracellular matrix (ECM), tissue metabolism, angiogenesis, and genetic disorders^7^. Therefore, understanding the relationship between breast cancer and obesity is crucial since this patient population faces particular diagnostic and therapeutic problems.

Distant metastasis is the primary cause of mortality among breast cancer patients; around 6% of patients already have distant metastases when initially diagnosed^8^. Tumor cells have a propensity to migrate to lymph node (LN) tissue because of the unique architecture of LN vessels which is a warning sign of metastatic tumors and an indicator for poorer OS for patients^9^. Research has discovered that focusing on metastatic LN might greatly improve the effectiveness of treatment on primary tumors, potentially serving as a crucial approach to enhance patient survival^10^. As such, it is critical to identify and characterize the LNM microenvironment.

For the cancer cells to continue proliferation, invasion, and metastasis to distant locations, they need reciprocal communication with cells in the intricate tissue surroundings. This communication can be facilitated by the release of regulatory molecules via membrane-enclosed particles known as extracellular vesicles (EVs) within the tumor microenvironment (TME) from cancer cells and stromal cells^11^. EVs are nanoscale structures enclosed by a lipid bilayer that play a pivotal role in communication between cells. EVs contain cargo molecules, including cytoplasmic proteins, lipids, and RNA, during their formation, as well as EV membrane-associated surface receptors^12^. These EVs have been identified in various body fluids including blood, urine, bile, breast milk, etc^12^. EVs encompass several types of vesicles, according to their size or how they are formed, including apoptotic bodies, microvesicles (MVs), and exosomes^13^. MVs typically measuring between 100 and 1000 nm in size are produced by the outward protrusion of the plasma membrane, and the other fraction is known as exosomes, which have a size range of 50 to 200 nm and originate from multivesicular bodies^13^. EVs produced from cancer cells are discharged into the TME and bloodstream and might accelerate tumor growth and spread of tumors by remodeling the surrounding microenvironment, promoting angiogenesis, inflammation, and creating the metastatic niche^14^. According to recent research, EVs in body fluids emerge as a candidate biomarker for detection and monitoring of cancer, because they are a less invasive and cost-effective alternative to tissue biopsy^15^. Therefore, the discovery of new non-invasive blood-based biomarkers for LNM may pave the way for breast cancer progression management.

Heparan sulfate proteoglycans (HSPGs) are a group of macromolecules expressed on the cell surface and within ECM^16^. These molecules are distinguished by the presence of one or more heparan sulfate (HS) chains that are covalently bonded, which is a form of glycosaminoglycans (GAGs)^16^. The HSPGs control a range of physiological functions, such as ECM assembly, cell-cell interactions, cell adhesion, and differentiation of cells^17^. According to the findings of previous studies, a significant association exists between the expression of certain HSPGs and the ability of breast cancer cells to metastasize and invade other tissues^18^. The transmembrane proteins known as syndecans (SDCs) are pivotal constituents of HSPGs. The family of SDCs includes four members, namely SDC1, SDC2, SDC3, and SDC4. Different studies have suggested that SDCs have a wide range of pathobiological activities in different tumor entities, including breast cancer^19^. SDCs regulate tumor angiogenesis, cell proliferation, migration, and invasion, which are linked to the advancement of tumors^20^. In addition, expression of SDCs correlates with tumor lymph angiogenesis in breast DCIS^21^. For example, in LNM the elevated expression level of SDC1 and E-cadherin suggests that these markers work together to promote tumor growth and invasion^22^. Moreover, different studies have demonstrated that SDC’s heparan sulfate, along with their cytoplasmic adaptor syntenin, regulate the process of EVs production^23,24^. In certain cell types and conditions, the SDC-syntenin-ALIX pathway significantly regulates a substantial portion of the vesicles that are released by cells^25^.

This study aimed to examine the expression patterns of SDC1, SDC2, and SDC4 in EVs isolated from peripheral blood of neoadjuvant chemotherapy-naïve obese breast cancer patients with negative LNM (nLNM) and positive LNM (pLNM) to discover their potential as new biomarkers for LNM.

## Results

### Clinicopathologic characteristics for breast cancer patients

We collected venous blood samples from 39 women diagnosed with primary breast cancer with an average age of (58.2 ± 1.9). All clinicopathological data of patients are summarized in Table 1. The patients were divided into two groups according to nodal metastasis status: 19 breast cancer patients with nLNM and 20 breast cancer patients with pLNM. All patients were obese with a BMI > 30 and neoadjuvant chemotherapy-naïve and no significant differences were observed between the two groups across all clinicopathological factors.

**Table 1:** The clinicopathological data of obese breast cancer patients with nLNM and pLNM.

Characteristic		nLNM (*n* = 19)	pLNM (*n* = 20)	*P*-value
Age (years)	Range	42–76	36–79	^a^*P* > 0.05
	Mean ± SEM	58.42 ± 2.75	58 ± 2.846	
	< 50	6 (31.58%)	6 (30%)	^b^*P* > 0.05
	≥ 50	13 (68.42%)	14 (70%)	
Body mass index (BMI)	Range	30.48–45.73	30.18–48.83	^a^*P* > 0.05
	Mean ± SEM	36.27 ± 1.031	36.76 ± 1.514	
Menopause status, n (%)	Premenopausal	7 (36.84%)	5 (25%)	^b^*P* > 0.05
	Postmenopausal	9 (47.37%)	11 (55%)	
	NA	3 (15.79%)	4 (20%)	
Family history, n (%)	Yes	4 (21.05%)	2 (10%)	^b^*P* > 0.05
	No	15 (78.95%)	17 (85%)	
	NA	-	1 (5%)	
Laterality, n (%)	Bilateral	-	1 (5%)	^b^*P* > 0.05
	Right	8 (42.11%)	9 (45%)	
	Left	11 (57.89%)	10 (50%)	
Tumor size (cm), n (%)	≤ 4	14 (73.68%)	17 (85%)	^b^*P* > 0.05
	> 4	5 (26.32%)	3 (15%)	
Tumor grade, n (%)	Grade I	2 (10.53%)	-	^b^*P* > 0.05
	Grade II	13 (68.42%)	16 (80%)	
	Grade III	3 (15.79%)	4 (20%)	
	NA	1 (5.26%)	-	
ER, n (%)	Negative	4 (21.05%)	1 (5%)	^b^*P* > 0.05
	Positive	14 (73.68%)	19 (95%)	
	NA	1 (5.26%)	-	
PR, n (%)	Negative	5 (26.32%)	2 (10%)	^b^*P* > 0.05
	Positive	13 (68.42%)	18 (90%)	
	NA	1 (5.26%)	-	
HER2, n (%)	Negative	14 (73.68%)	15 (75%)	^b^*P* > 0.05
	Equivocal (non-amplified)	5 (26.32%)	5 (25%)	
Stages, n (%)	Early stage			^**b**^***P*** **< 0.05**
	IA	10 (52.63%)	-	
	IB	-	1 (5%)	
	IIA	7 (36.84%)	3 (15%)	
	IIB	2 (10.53%)	8 (40%)	
	IIIA	-	1 (5%)	
	Late stage			
	IIIA	-	3 (15%)	
	IIIB	-	1 (5%)	
	IIIC	-	3 (15%)	
Lymph node status, n (%)	N0	19 (100%)	-	^**b**^***P*** **< 0.05**
	N1	-	13 (65%)	
	N2	-	4 (20%)	
	N3	-	3 (15%)	
Tumor size, n (%)	T1	10 (52.63%)	6 (30%)	^b^*P* > 0.05
	T2	7 (36.84%)	12 (60%)	
	T3	2 (10.53%)	1 (5%)	
	T4	-	1 (5%)	
Molecular subtypes	Luminal A	8 (42.11%)	8 (40%)	^b^*P* > 0.05
	Luminal B	9 (47.37%)	9 (45%)	
	Triple-negative	2 (10.53%)	1 (5%)	
	NA	-	2 (10%)	
Ki-67% n (%)	Low (< 20%)	9 (47.37%)	8 (40%)	^b^*P* > 0.05
	High (≥ 20%)	7 (36.84%)	9 (45%)	
	NA	3 (15.79%)	3 (15%)	

^a^*P-* value for one way ANOVA test.

^b^*P-* value for Chi-square test.

**Fig. 1:**
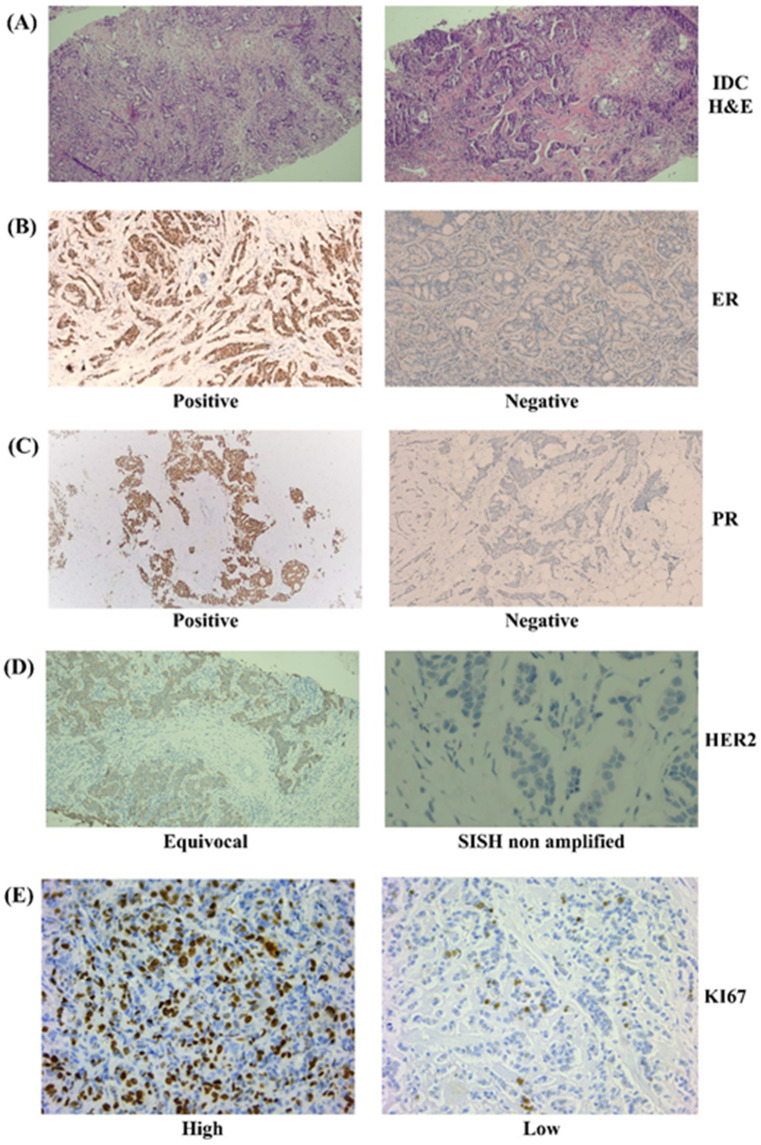
Examination of breast cancer tissue samples using histopathological and immunohistochemical (IHC) staining. **(A)** Hematoxylin and Eosin (H&E) staining showing the morphological features of breast cancer. IDC: invasive ductal carcinoma (magnification 100×). **(B)** IHC staining demonstrating positive estrogen receptor (ER) expression (left) and a lack of ER expression (right) (magnification 100×). **(C)** IHC staining revealing progesterone receptor (PR) positivity (left) and PR negativity (right)(magnification 100×). **(D)** IHC staining showing an equivocal level of human epidermal growth factor receptor 2 (HER2) (left) (magnification 100×) and silver in situ hybridization (SISH) indicating negative HER2 expression (right) (magnification 400×). **(E)** IHC staining illustrating high Ki-67 expression (> 20%) (left) and low Ki-67 expression (< 20%) (right)(magnification 200×).

Hematoxylin and Eosin (H&E)-stained sections from the primary tumor revealed distinct and well-separated patterns of spatial morphological heterogeneity (Fig. 1). The pathologic stage was determined using the tumor-node-metastasis system as outlined in the American Joint Committee on Cancer (AJCC) 7^th^-edition guidelines^26^. Tumor grading was performed by analyzing tissue morphology, in accordance with the WHO Classification of Breast Tumors (5^th^ Edition, 2019). In our study, cases were grouped into three unique molecular subgroups based on the expressions of ER, PR, HER2, and Ki-67, as depicted in Fig. 1. Luminal A cancers are identified by the presence of ER and/or PR, the absence of HER2, and a Ki-67 expression level below 20%. Luminal B (HER2-negative) cancers are characterized by ER positivity, HER2 negativity, and either high Ki-67 expression or low/absent PR expression. Triple-negative breast cancer is defined by the absence of ER, PR, and HER2^27^.

### Characterization of the isolated EVs

**Fig. 2:**
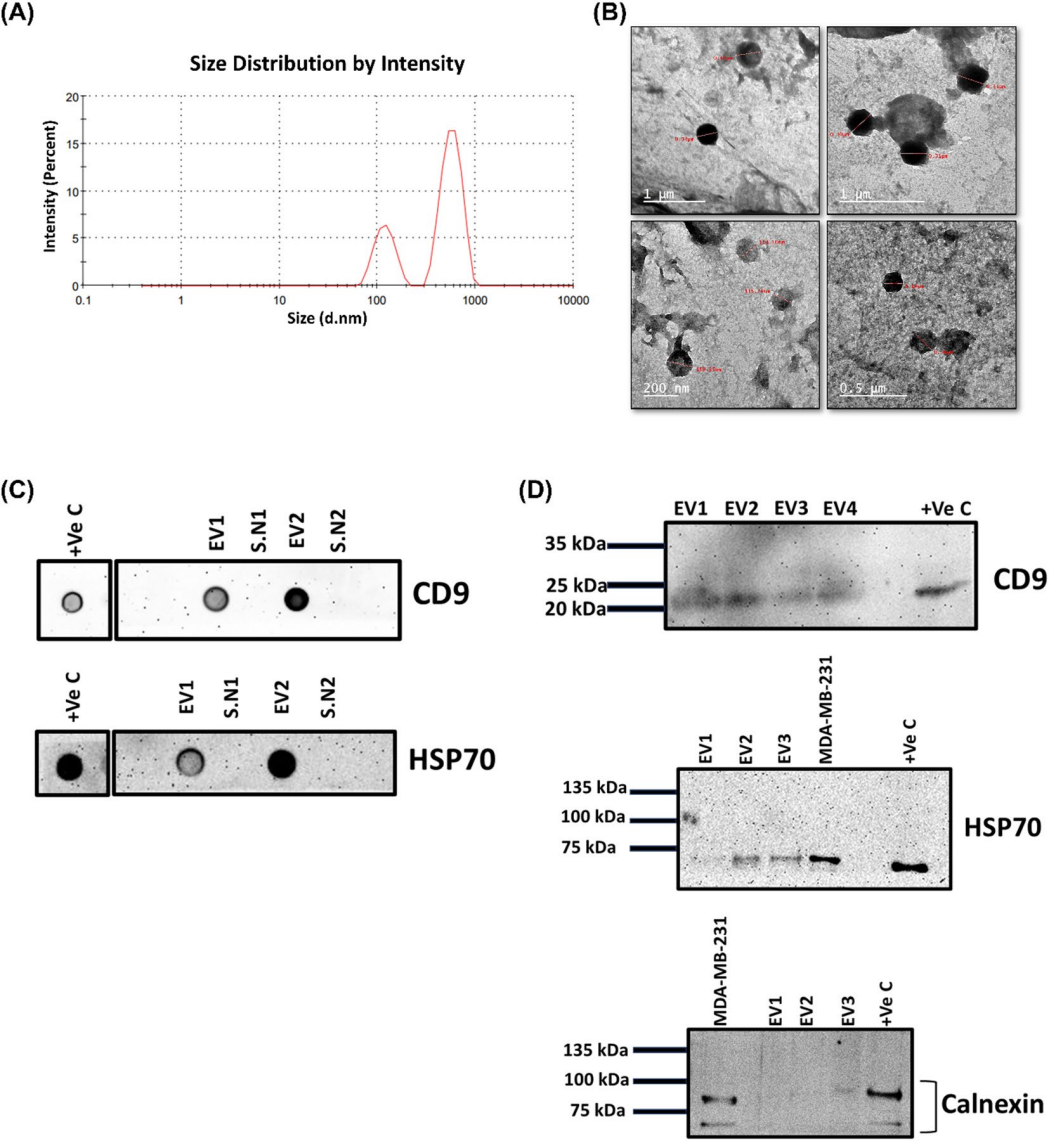
Characterization of plasma extracellular vesicles (EVs) isolated from obese breast cancer patients. **(A)** Size distribution of EVs determined by Dynamic Light Scattering (DLS). **(B)** Transmission Electron Microscopy (TEM) demonstrates the size of EVs with an average > 100 nm. **(C)** Dot blot for the presence of the EV markers CD9 and HSP70. **(D)** Western blot for EV marker CD9 and HSP70 and non-EV marker Calnexin. The membranes were cut before being probed with primary antibodies targeting CD9, HSP70, and Calnexin. The uncropped membranes with varying exposure times are shown in Supplementary Fig. S1a-e.

To confirm the successful isolation of EVs, we used different approaches including dynamic light scattering (DLS), transmission electron microscopy (TEM), dot and western blots. Using DLS, we observed two EV populations: one population with a size distribution of 577.4 nm with intensity 72.4% and the other population with a size distribution 120.5 nm with intensity 27.6% (Fig. 2A). The calculated Polydispersity Index (PDI) was 0.572. TEM images revealed EVs with a circular shape, ensuring that the isolated EVs were intact with the average size > 100 nm as revealed by DLS (Fig. 2B). The presence of the established markers of EVs CD9 and HSP70 were further confirmed by dot and western blots (Fig. 2C&D). The endoplasmic reticulum protein Calnexin as EV-negative marker has not been detected in the isolated EVs (Fig. 2D), further ensuring the purity of the isolated EVs and not being contaminated with cellular proteins. Although the 21,000 × g centrifugation step is designed to enrich larger vesicles known as MVs, the presence of smaller particles suggests co-isolation of other EV subtypes. Therefore, we describe our isolate as “microvesicles-enriched extracellular vesicles (MV-enriched EVs)”.

### High SDC2 and fibronectin (FN) expressions in MV-enriched EVs derived from breast cancer patients with pLNM

**Fig. 3:**
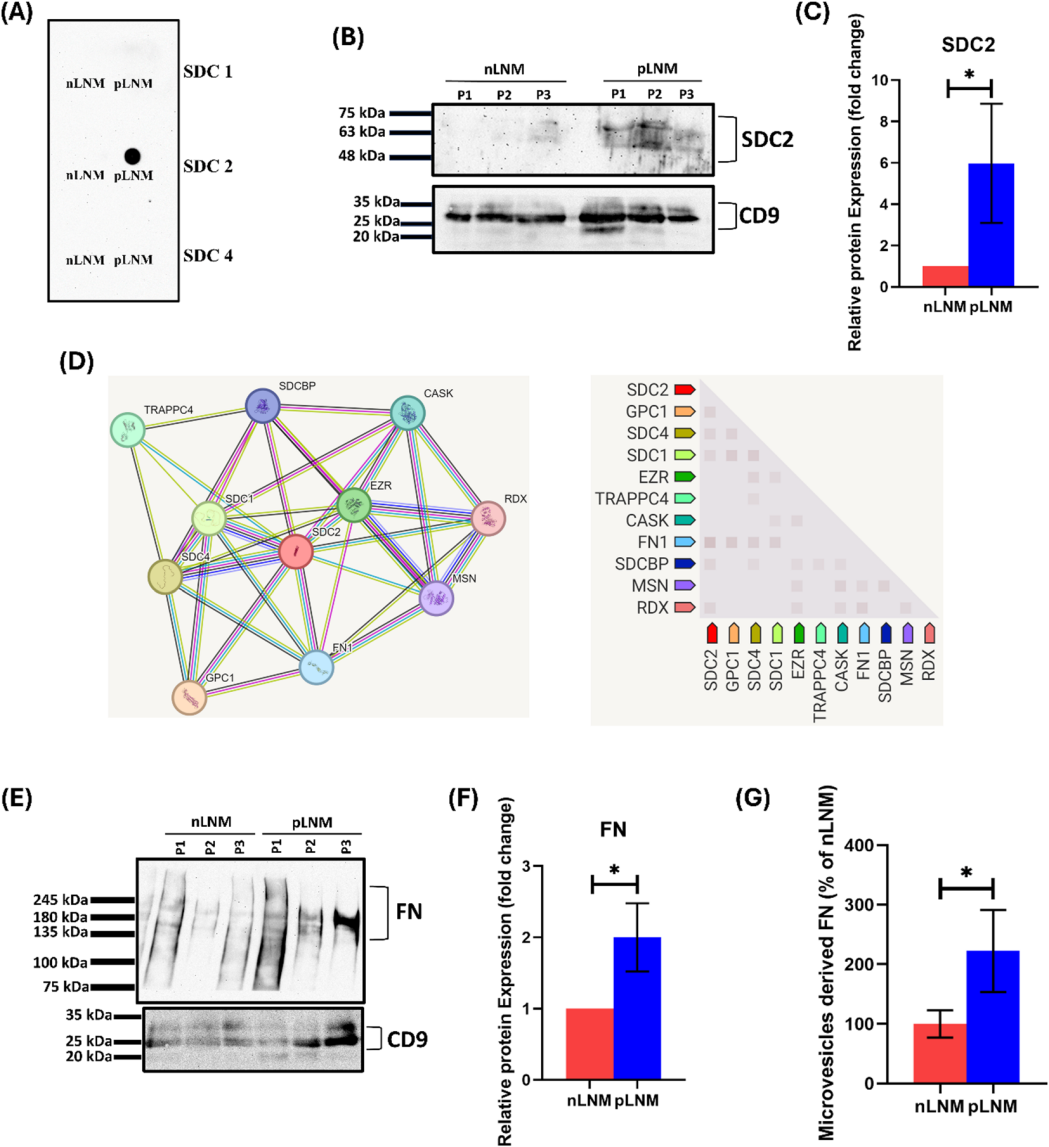
Expression of MV-enriched EV-derived SDC2 and FN. **(A)** Dot blot of SDC1, SDC2, and SDC4 derived from MV-enriched EVs of both nLNM and pLNM obese breast cancer patients showing the presence of SDC2 only in pLNM **(B)** Western blot of SDC2 isolated from MV-enriched EVs of plasma samples showing an increase in the expression of SDC2 in pooled samples of patients with pLNM (*n* = 3) compared with patients with nLNM (*n* = 3). P: pooled samples. **(C)** The intensity of the bands was quantified and normalized using CD9 as the loading control. *P < 0.05. **(D)** Protein-protein interaction (PPI) network of SDC2 using STRING database v.11 (http://string-db.org/) (accessed on 25 August 2024) indicating that SDC2 is co-expressed with FN with the highest score of 0.146. **(E)** Western blot of MV-enriched EV-derived FN reveals an upregulation in the expression of FN in pooled samples of patients with pLNM (*n* = 3) compared to nLNM patients (*n* = 3). P: pooled samples. **(F)** The band intensity was quantified and normalized to CD9, which served as the loading control. *P < 0.05. **(G)** FN levels assessed by ELISA (enzyme-linked immunosorbent assay) in plasma-derived MV-enriched EVs in nLNM and pLNM obese breast cancer patients, *P < 0.05. The membranes were cut before being probed with primary antibodies targeting SDC2, FN, and CD9. The uncropped membranes with varying exposure times are shown in Supplementary Fig. S2a-e.

Since SDC1, SDC2, and SDC4 are involved in the growth of breast carcinomas^19^ and EVs biogenesis^23^, we used dot blot to uncover whether the isolated MV-enriched EVs contained SDC1, SDC2, and SDC4. Our results showed that MV-enriched EVs isolated from obese breast cancer patients with pLNM contained higher SDC2, but not SDC1 and SDC4, compared to those with nLNM (Fig. 3A). We next validated this finding by western blot. Our data analysis demonstrated that MV-enriched EVs contained higher SDC2 in obese breast cancer patients with pLNM than in those with nLNM (*P* < 0.05) (Fig. 3B&C).

The dynamic interaction between SDC2 and FN contributes to the metastatic properties of cancer cells^28^, a connection that is further evidenced by Search Tool for the Retrieval of Interacting Genes (STRING) network analysis, where FN was the most SDC2-interacting protein partner with co-expression score of 0.146 **(**Fig. 3D**)**. In addition, FN was found to be a component of MVs^29^ and plays a role in modulating EV-cell interactions^30^. Therefore, we explored the expression pattern of FN in MV-enriched EVs. Our western blot data uncovered that MV-enriched EVs contained higher FN in obese breast cancer patients with pLNM than in those with nLNM (*P* < 0.05) **(**Fig. 3E**&F)**.

We further quantified the levels of FN derived from MV-enriched EVs in both nLNM and pLNM obese breast cancer patients using ELISA. Our data indicate that the average concentration of MV-enriched EV-derived FN was significantly higher by approximately 2.5-fold in obese breast cancer patients with pLNM (21.57 ± 11.78 ng/mL) than in those with nLNM (9.03 ± 4.244 ng/mL) (*P* < 0.05) **(**Fig. 3G**)**.

### Low *SDC2* and *FN* mRNA expressions in carcinoma tissues of breast cancer patients with pLNM

**Fig. 4:**
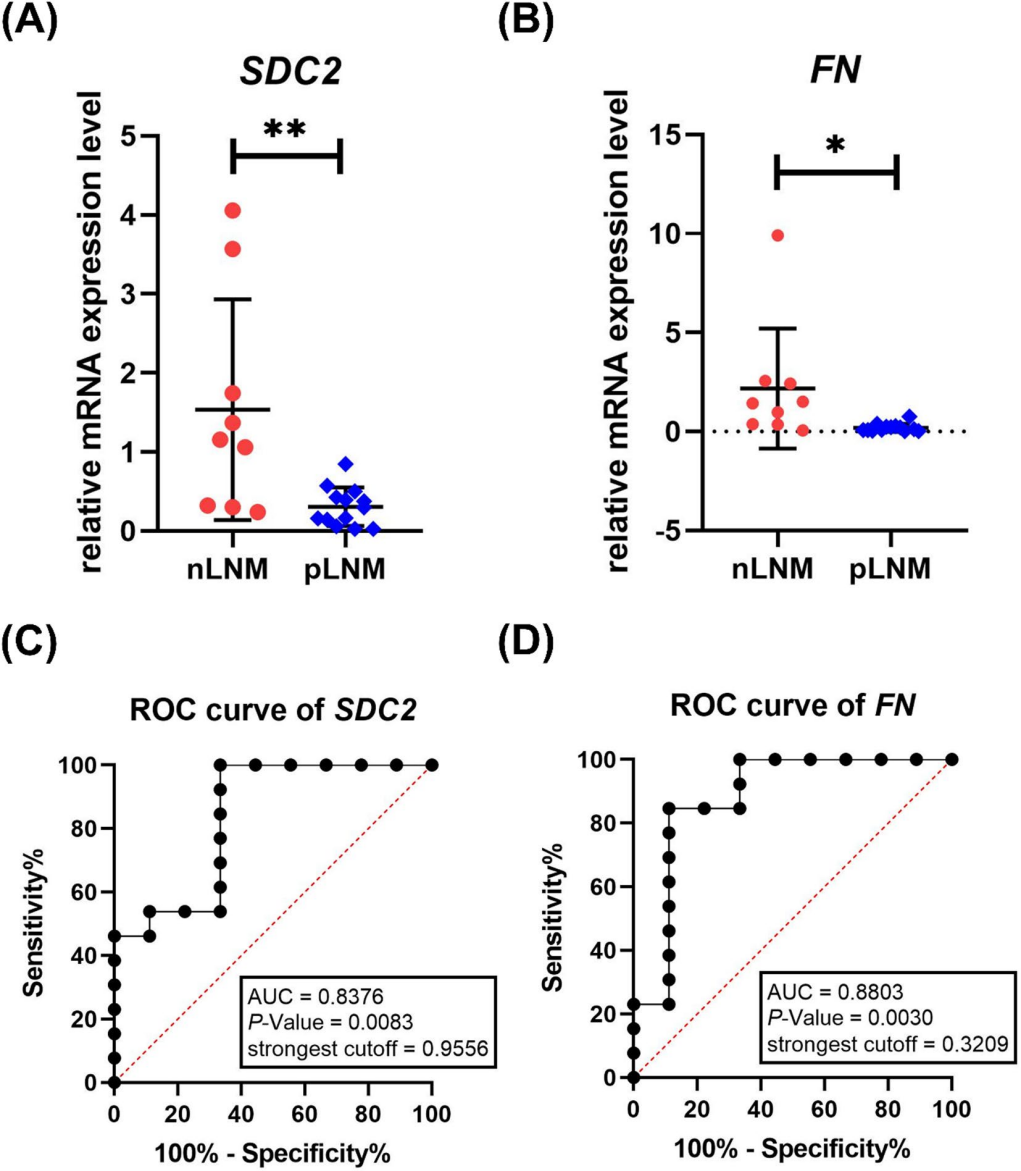
Expression and diagnostic significance of *SDC2* and *FN* in tumor tissues of obese breast cancer patients with pLNM compared to those with nLNM. **(A)** qPCR analysis showing a significant downregulation of *SDC2* expression in tumor tissues of the pLNM group (*n* = 13) relative to the nLNM group (*n* = 9). **P < 0.01. **(B)** qPCR results demonstrate a notable decrease in *FN* expression levels in tumor tissues of the pLNM group (*n* = 13) when compared to those in the nLNM group (*n* = 9). *P < 0.05. **(C)** Receiver operating characteristic (ROC) curve was constructed using *SDC2* expression levels to differentiate between nLNM and pLNM obese breast cancer patients. The area under the curve (AUC) = 0.8376, **P < 0.01. **(D)** ROC curve was generated to analyze expression levels of *FN* in distinguishing pLNM from nLNM obese breast cancer patients. AUC = 0.8803, **P < 0.01.

qRT-PCR was employed to quantify the transcript levels of *SDC2* and *FN* in tumor tissue specimens from breast cancer patients with nLNM and pLNM. Our results demonstrated a marked decrease in the mRNA expression of *SDC2* (*P* < 0.01) and *FN* (*P* < 0.05) in tumor tissue samples from pLNM breast cancer patients compared to those with nLNM (Fig. 4A&B). Furthermore, Receiver operating characteristic (ROC) analysis revealed that *SDC2* had an area under the curve (AUC) of 0.8376 (*P* < 0.01), with a sensitivity of 100% and a specificity of 66.67%, and *FN* achieved an AUC of 0.8803 (*P* < 0.01), with a sensitivity of 84.62% and a specificity of 88.89% for distinguishing pLNM from nLNM patients (Fig. 4C&D). However, the combination of *SDC2* and *FN* did not significantly impact the AUC value, indicating no additive or synergistic effect on the predictive performance.

### Functional and pathway enrichment analyses of SDC2 and FN

To better understand the biological roles of SDC2 and FN, we performed functional and Kyoto Encyclopedia of Genes and Genomes (KEGG) pathway enrichment analyses using the STRING and the Database for Annotation, Visualization, and Integrated Discovery (DAVID) online tool **(**Table 2**)**. Our analysis revealed that the Biological Processes (BP) of SDC2 and FN were primarily engaged in cell migration (*P* < 0.05). In terms of Cellular Components (CC), SDC2 was mainly enriched in cell surface (*P* < 0.01) and plasma membrane (*P* < 0.05), and FN was enriched in plasma membrane (*P* < 0.05), extracellular exosomes (*P* < 0.05), and extracellular membrane-bounded organelles (*P* < 0.05). In the context of Molecular Function (MF), SDC2 and FN were primarily involved in protein binding (*P* < 0.001). KEGG pathway analysis revealed that both SDC2 and FN were enriched in the proteoglycans in cancer pathway (*P* < 0.0001). In addition, SDC2 was associated with cell adhesion (*P* < 0.001), while FN was enriched in ECM-receptor interaction (*P* < 0.001).

**Table 2:** Functional and pathway enrichment analyses of SDC2 and FN.

Category	Term description	Gene count	Strength	*P*-value	Matching proteins
**STRING database**					
Biological Process	Positive regulation of extracellular exosome assembly	2	3.39	0.0073	SDC4,SDC1
	Cell migration	4	1.34	0.022	SDC2,FN1,SDC4,SDC1
	Positive regulation of exosomal secretion	2	2.82	0.022	SDC4,SDC1
	Wound healing	3	1.64	0.0447	FN1,SDC4,SDC1
Cellular Component	Golgi lumen	3	2.14	0.001	SDC2,SDC4,SDC1
	Lysosomal lumen	3	2.18	0.001	SDC2,SDC4,SDC1
Subcellular Localization	Golgi lumen	3	2.16	0.00093	SDC2,SDC4,SDC1
	Lysosomal lumen	3	2.22	0.00093	SDC2,SDC4,SDC1
	Extracellular exosome	3	1.54	0.0235	FN1,SDC4,SDC1
	Extracellular membrane-bounded organelle	3	1.49	0.0253	FN1,SDC4,SDC1
KEGG Pathways	Proteoglycans in cancer	4	2.01	3.32E-06	SDC2,FN1,SDC4,SDC1
	ECM-receptor interaction	3	2.23	6.38E-05	FN1,SDC4,SDC1
	Cell adhesion molecules	3	2.03	0.00013	SDC2,SDC4,SDC1
	Fluid shear stress and atherosclerosis	3	2.06	0.00013	SDC2,SDC4,SDC1
	Malaria	2	2.33	0.0023	SDC2,SDC1

Category	Term description	Gene count	Strength	FDR	Matching proteins
**DAVID online tool**					
Biological Process	Cell migration	3	52.7	0.0006	SDC4, SDC2, SDC1
	Positive regulation of extracellular exosome assembly	2	2466.8	0.00061	SDC4, SDC1
	Positive regulation of exosomal secretion	2	657.8	0.0023	SDC4, SDC1
	Ureteric bud development	2	266.7	0.0056	SDC4, SDC1
	Wound healing	2	126.5	0.012	SDC4, FN1
Cellular Component	Lysosomal lumen	3	159.8	0.000065	SDC4, SDC2, SDC1
	Golgi lumen	3	146.4	0.000078	SDC4, SDC2, SDC1
	Cell surface	3	23.8	0.0029	SDC4, SDC2, SDC1
	Plasma membrane	4	3.7	0.019	SDC4, SDC2, FN1, SDC1
	Extracellular exosome	3	6.9	0.033	SDC4, FN1, SDC1
	Endoplasmic reticulum lumen	2	33.6	0.044	SDC2, FN1
	Collagen-containing extracellular matrix	2	25.6	0.057	SDC2, FN1
Molecular Function	Identical protein binding	4	11.1	0.00073	SDC4, SDC2, FN1, SDC1
KEGG Pathways	Proteoglycans in cancer	4	43.3	0.000012	SDC4, SDC2, FN1, SDC1
	Cytoskeleton in muscle cells	4	38.1	0.000018	SDC4, SDC2, FN1, SDC1
	ECM-receptor interaction	3	74.5	0.0003	SDC4, FN1, SDC1
	Fluid shear stress and atherosclerosis	3	47	0.00075	SDC4, SDC2, SDC1
	Cell adhesion molecules	3	42.2	0.00093	SDC4, SDC2, SDC1
	Malaria	2	88.4	0.017	SDC2, SDC1

### SDC2 and FN expression in breast cancer patients

**Fig. 5:**
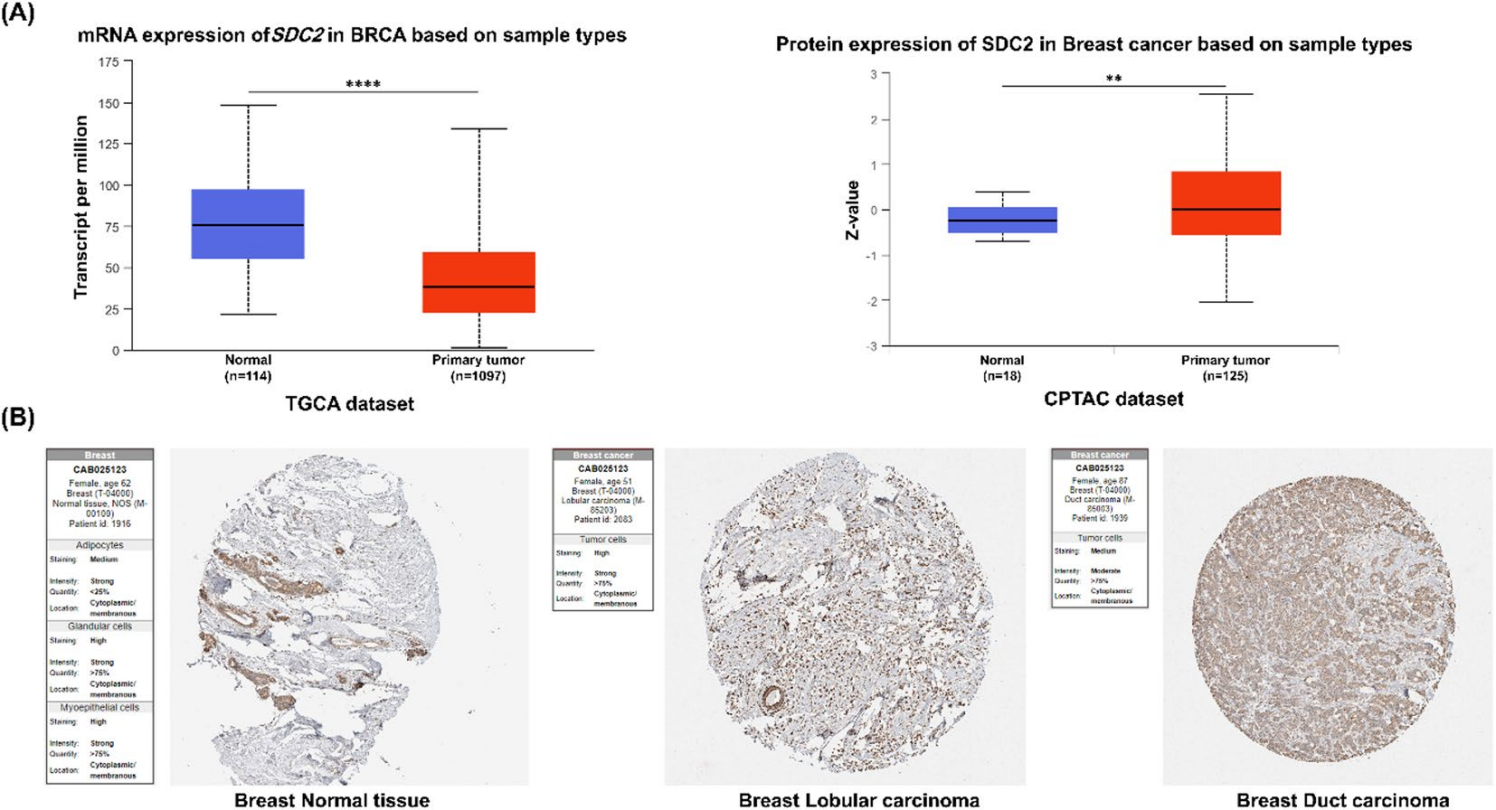
Expression of SDC2 in breast cancer using online databases. **(A)** mRNA expression of *SDC2* was downregulated in breast carcinoma tissues compared with normal tissues, whereas SDC2 protein level was upregulated in the UALCAN online database (http://ualcan.path.uab.edu/) (accessed on 25 August 2024). **(B)** Representative immunohistochemical (IHC) images of SDC2 expression selected from the Human Protein Atlas (HPA) (https://www.proteinatlas.org/*)* (accessed on 25 August 2024). **P < 0.01; ****P < 0.0001.

**Fig. 6:**
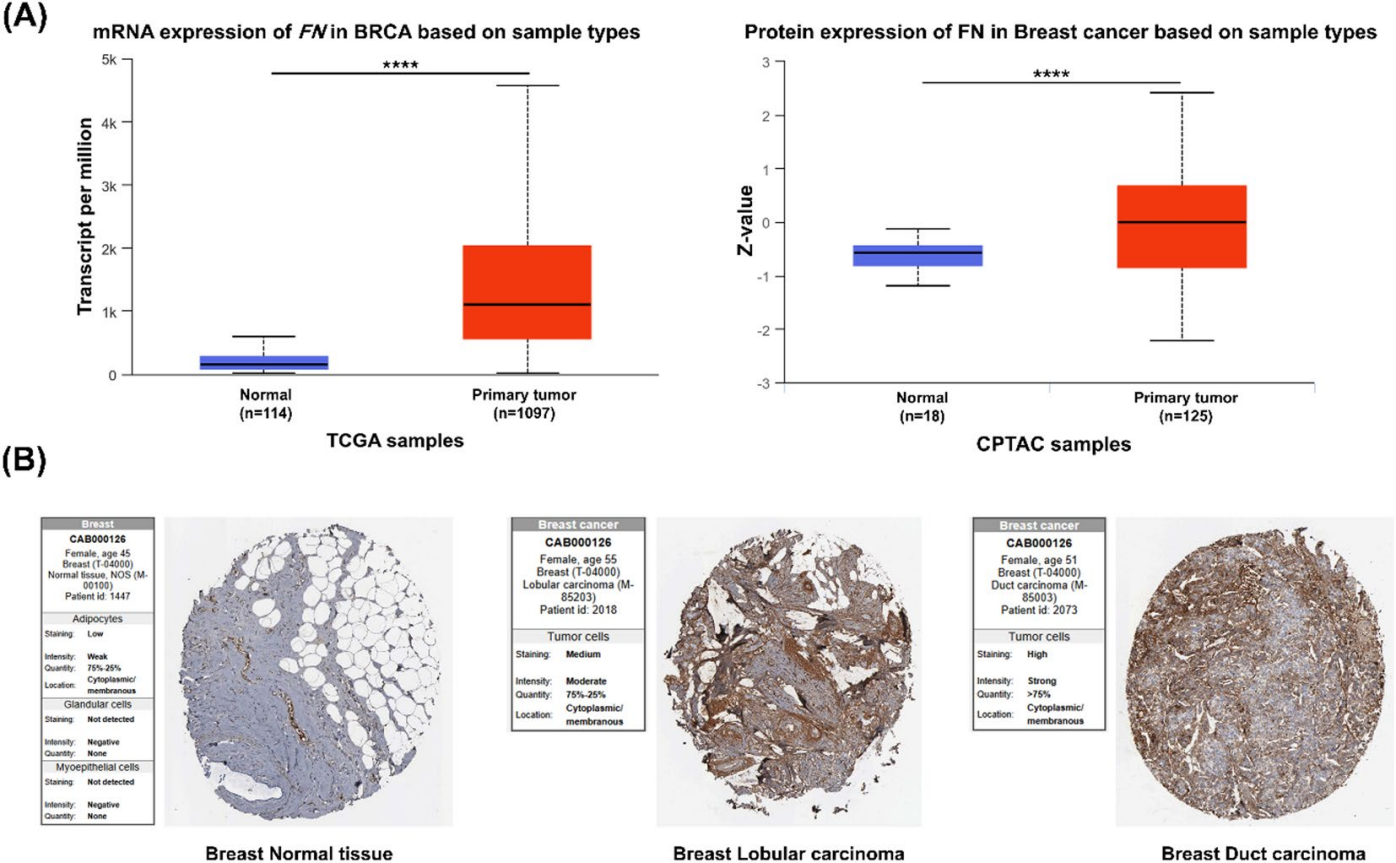
Expression analysis of FN in breast cancer tissues using online databases. **(A)** mRNA and protein expression levels of FN were upregulated in breast cancer tissues compared to normal tissues analyzed using the UALCAN database (http://ualcan.path.uab.edu/) (accessed on 13 June 2025). **(B)** Representative immunohistochemical (IHC) images of FN expression selected from the Human Protein Atlas (HPA) (https://www.proteinatlas.org/) (accessed on 13 June 2025). ****P < 0.0001.

It was reported that dysregulated expression of SDC2 occurs during the progression of breast cancer, promoting the growth and migration of the tumors^31^. Therefore, we first analyzed the expression of *SDC2* mRNA and protein in breast carcinoma tissues using the UALCAN online database with respect to the matched normal tissues. The analysis demonstrated a significant downregulation of *SDC2* mRNA expression in breast carcinoma tissues (*P* < 0.0001), whereas the SDC2 protein was found at higher levels in these cancer tissues compared to that found in normal tissues (*P* < 0.01). (Fig. 5A). These data were further corroborated using the Human Protein Atlas (HPA), where increased immunoreactivity of the SDC2 protein was detected in breast carcinoma tissues relative to normal tissues (Fig. 5B). We assessed FN expression patterns using the UALCAN online database. The results demonstrated a significant increase in both *FN* mRNA and protein levels in primary breast tumors compared to normal tissues (*P* < 0.0001) (Fig. 6A). These data were confirmed by immunohistochemical (IHC) analysis, which revealed minimal FN staining in normal breast tissue, while moderate to high cytoplasmic and membranous FN expression was observed in both lobular and ductal carcinoma tissues (Fig. 6B).

### Association of *SDC2* and *FN* mRNA expression with nodal metastasis status and cancer stages

**Fig. 7:**
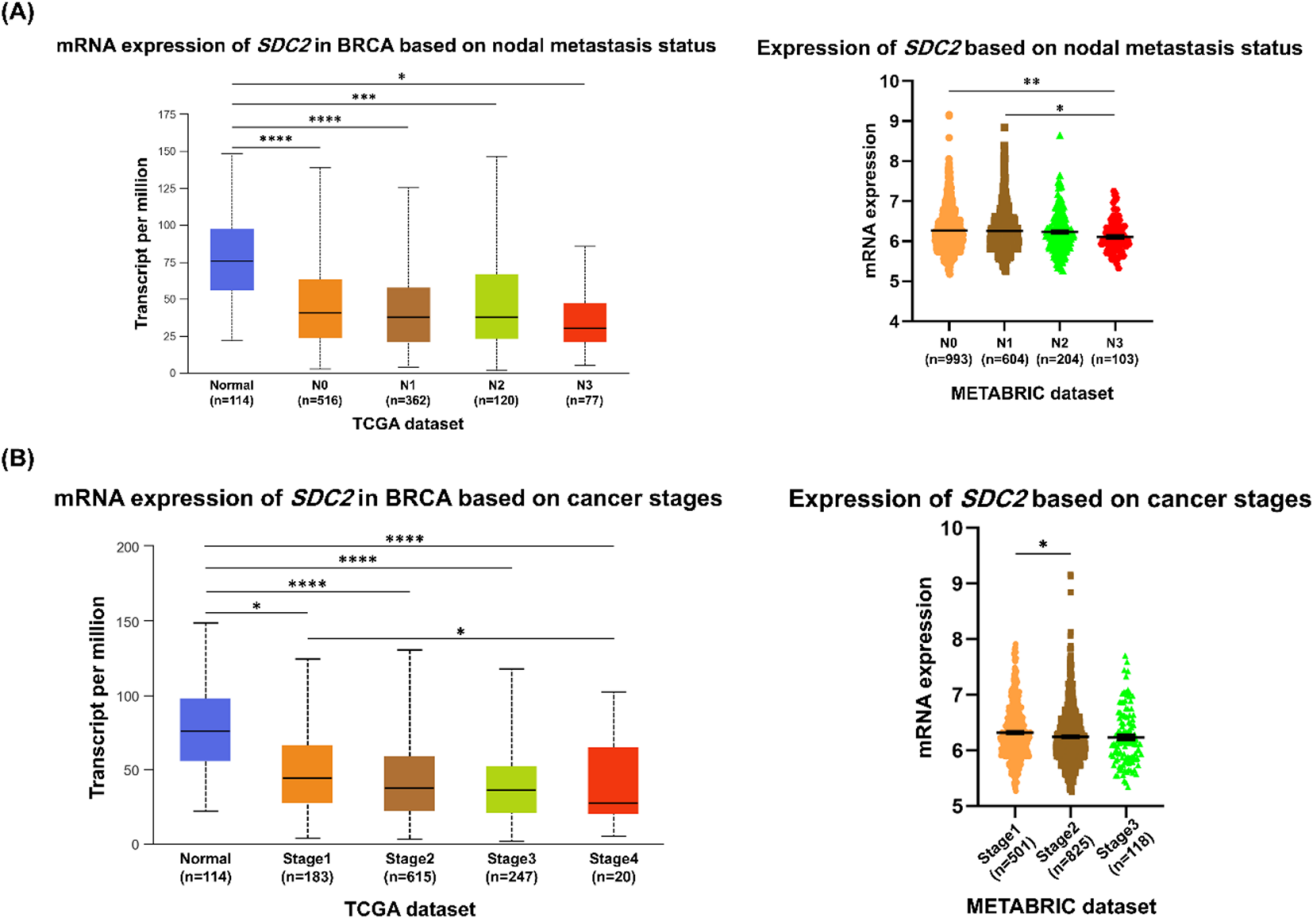
mRNA expression level of *SDC2* in breast carcinoma tissues compared with normal tissues based on **(A)** nodal status and **(B)** clinical stage, according to METABRIC (https://www.cbioportal.org/*)* (accessed on 25 August 2024) and UALCAN database (http://ualcan.path.uab.edu/) (accessed on 25 August 2024). *P < 0.05; **P < 0.01; ***P < 0.001; ****P < 0.0001.

We investigated *SDC2* and *FN* mRNA expression in breast cancer according to nodal metastasis status and cancer staging using the UALCAN and cBioPortal online databases. *SDC2* mRNA expression was notably downregulated in breast carcinoma tissues stratified by nodal metastasis status (N0, N1, N2, and N3), which aligned with our qPCR results in breast cancer tissue samples **(**Fig. 7A**)** and staging (I, II, III, and IV) relative to normal tissues **(**Fig. 7B**)**.

**Fig. 8:**
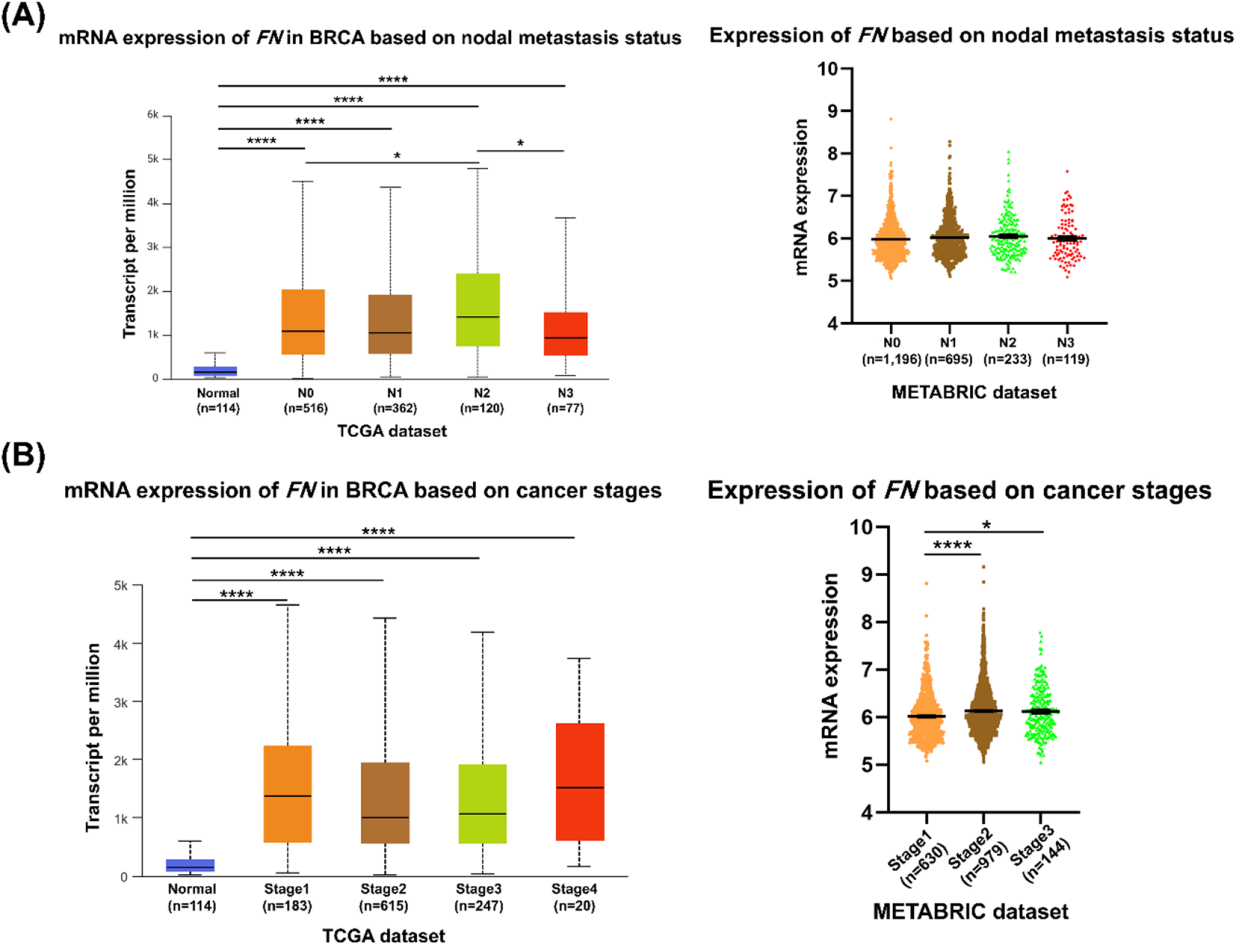
mRNA expression level of *FN* in breast carcinoma tissues compared with normal tissues based on **(A)** nodal status and **(B)** clinical stage according to METABRIC (https://www.cbioportal.org/) (accessed on 13 June 2025) and UALCAN database (http://ualcan.path.uab.edu/) (accessed on 13 June 2025). *P < 0.05; ****P < 0.0001.

TCGA datasets indicate that *FN* mRNA expression levels were significantly increased in breast cancer patients with N2 relative to those with N0 (*P* < 0.05). However, this level decreased in breast cancer patients with N3 in comparison with those with N2 (*P* < 0.05) (Fig. 8A). The METABRIC dataset did not reveal any significant differences among breast cancer patients with pLNM compared with nLNM. According to cancer staging, in the TCGA dataset, *FN* mRNA expression level was overall significantly increased among breast cancer patients with different stages compared to normal tissues, with higher levels in stage II (*P* < 0.0001) and III (*P* < 0.05) than in stage I according to METABRIC dataset (Fig. 8B).

### Prognostic value of SDC2 and FN expression in breast cancer

**Fig. 9:**
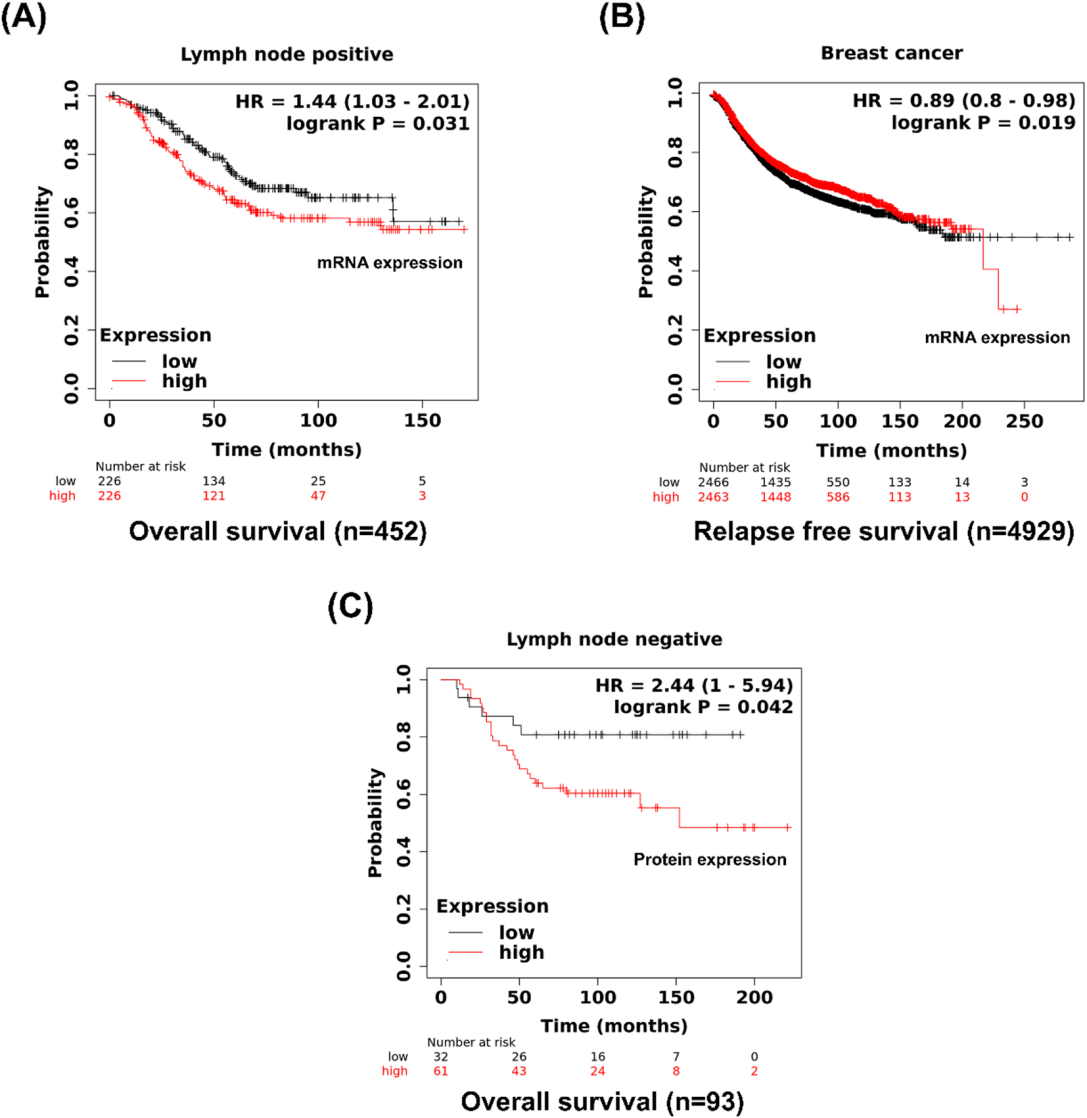
Kaplan-Meier overall survival (OS) or relapse-free survival (RFS) curves (http://kmplot.com/analysis/*)* (accessed on 25 August 2024) are plotted for mRNA or protein SDC2 expression levels in all breast cancer patients (*n* = 4929) or breast cancers with negative (*n* = 93) or positive (*n* = 452) lymph node.

**Fig. 10:**
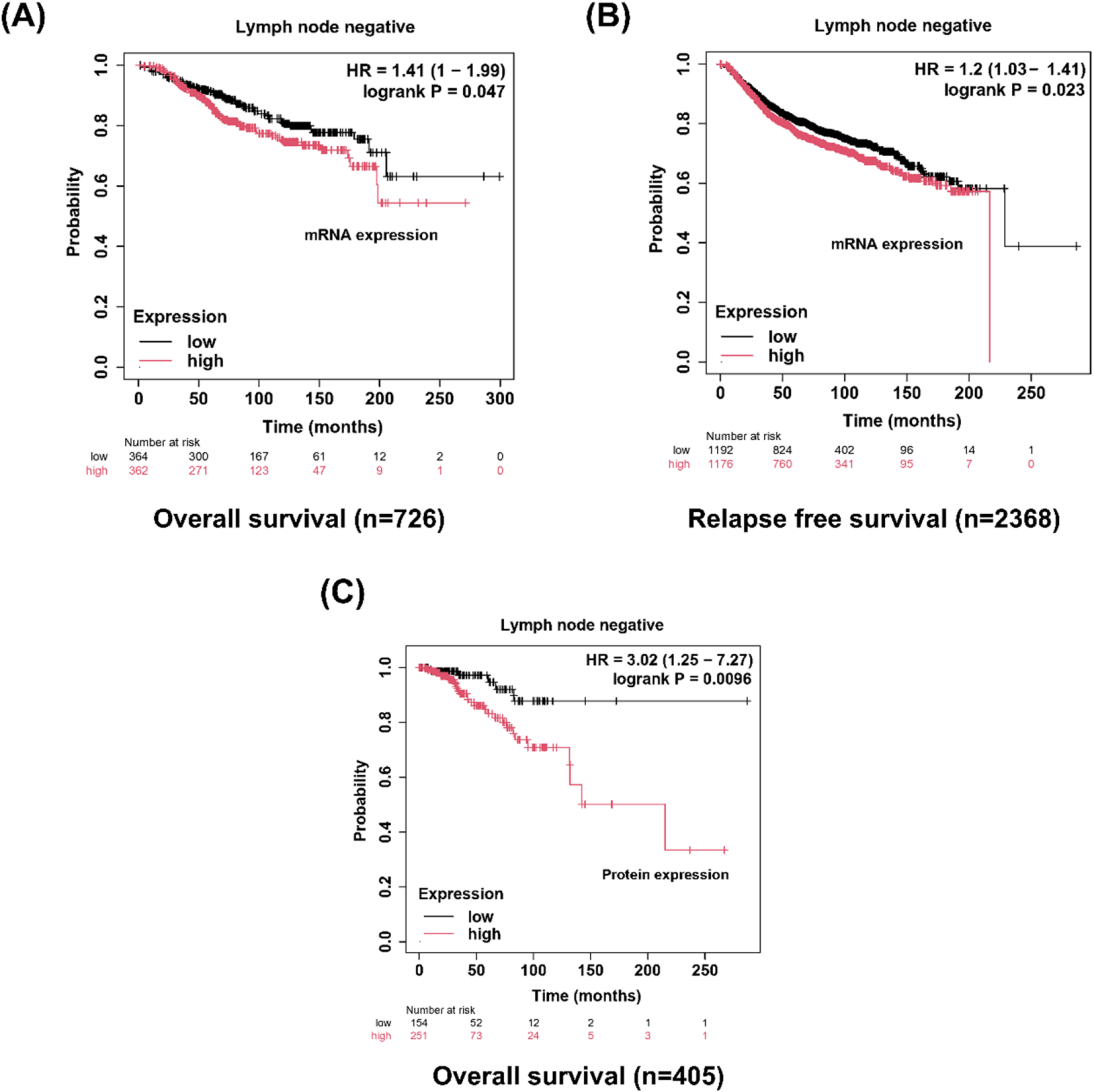
Kaplan–Meier overall survival (OS) and relapse-free survival (RFS) curves (http://kmplot.com/analysis/) (accessed on 13 June 2025) for mRNA and protein FN expression levels in breast cancer patients with negative lymph node status. High *FN* mRNA expression was associated with decreased OS (*n* = 726) and shorter RFS (*n* = 2368), while elevated FN protein levels were significantly linked to poorer OS (*n* = 405).

We next employed the Kaplan–Meier Plotter to explore the prognostic significance of SDC2 and FN expression (Fig. 9). The results indicated that breast cancer patients with pLNM who had lower levels of *SDC2* mRNA expression experienced better OS (*P* < 0.05, *n* = 452) (Fig. 9A). High mRNA levels of *SDC2* showed shorter relapse-free survival (RFS) in all breast cancer patients (*P* < 0.05, *n* = 4929) (Fig. 9B). Moreover, high SDC2 expression at the protein level was associated with worse OS in breast cancer patients with nLNM (*P* < 0.05, *n* = 93) (Fig. 9C). On the other hand, the mRNA and protein levels of FN in breast cancer patients with nLNM were significantly associated with poor prognosis. High *FN* mRNA expression was significantly associated with decreased OS (*P* < 0.05, *n* = 726) and a shorter RFS (*P* < 0.05, *n* = 2368) (Fig. 10A&B). Moreover, high protein level of FN was markedly associated with reduced OS (*P* < 0.001, *n* = 405) (Fig. 10C).

### *SDC2* and *FN* as predictive markers for treatment response in breast cancer

**Fig. 11:**
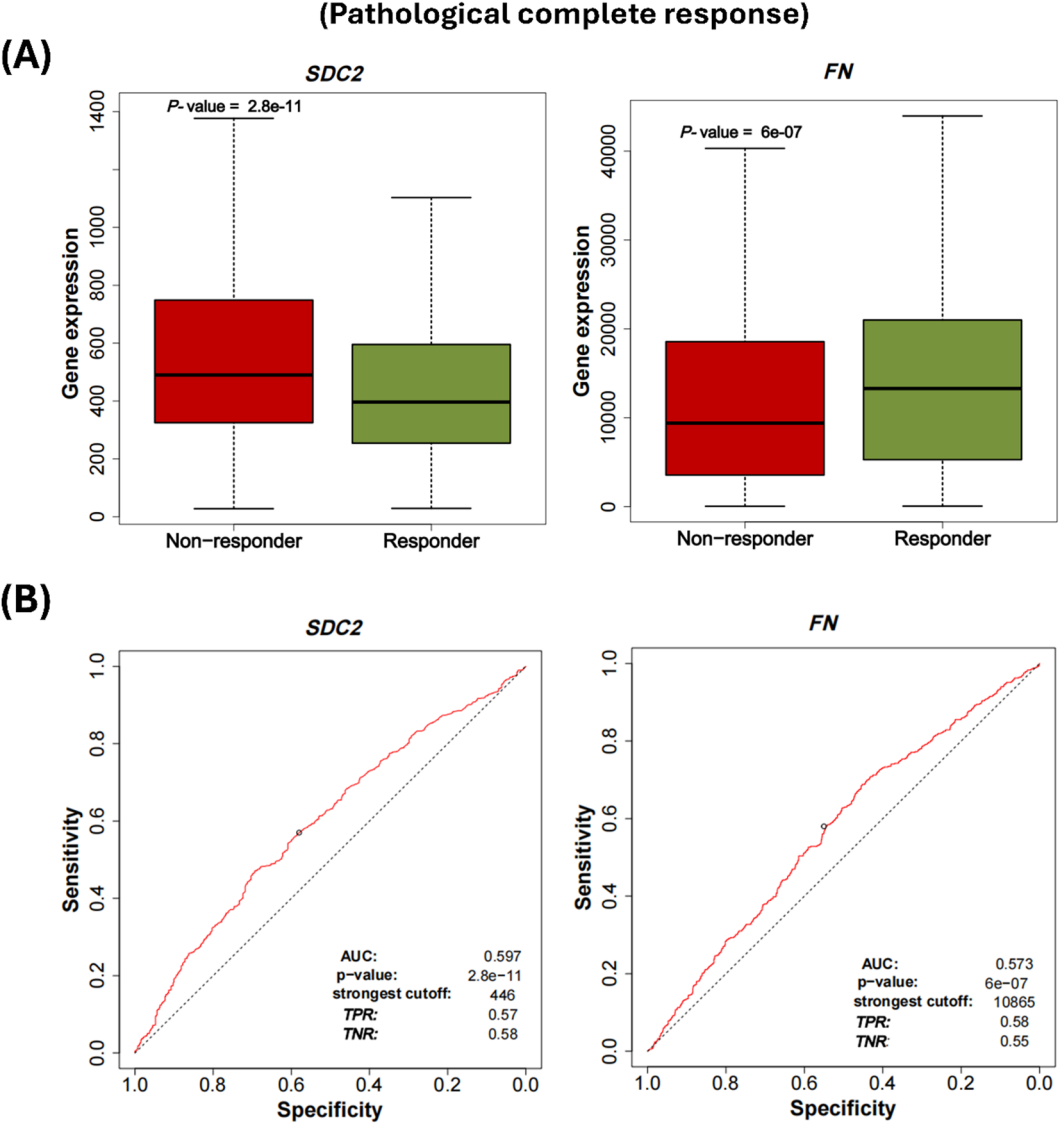
Analysis of *SDC2* and *FN* as a predictive biomarker for chemotherapy treatment response in breast cancer using ROC plotter database (http://www.rocplot.org/) (accessed on 24 October 2024). **(A)** Boxplots comparing the gene expression of *SDC2* and *FN* in breast cancer patients between non-responder (*n* = 1100) and responder (*n* = 532) to chemotherapy. **(B)** ROC curves demonstrating the sensitivity and specificity of *SDC2* and *FN* in predicting patient response to chemotherapy treatment. The AUC, corresponding *P*-values, and the strongest and optimal cutoffs are shown. ROC: receiver operating characteristic, AUC: area under the curve, TPR: true positive rate, TNR: true negative rate.

We used the ROC Plotter web tool (http://www.rocplot.org/) (accessed on 24 October 2024) to analyze the link between *SDC2* and *FN* expression and resistance to chemotherapy based on transcriptome-level data of breast cancer. Patients with high *SDC2* levels responded less to therapy than those with low levels (*P* < 0.0001), while a positive response to chemotherapy was significantly associated with higher levels of *FN* expression (*P* < 0.0001) **(**Fig. 11A**)**. As depicted in Fig. 11B, AUC for *SDC2* and *FN* were 0.597 (*P* < 0.0001) and 0.573 (*P* < 0.0001), respectively, suggesting that *SDC2* and *FN* may serve as effective biomarkers for differentiating between chemotherapy responders and non-responders.

Next, we examined the association between the expression levels of *SDC2* and *FN* genes and the therapeutic response of breast cancer patients to different chemotherapy treatments such as Taxane, Anthracycline, Ixabepilone, Cyclophosphamide, Methotrexate, and Fluorouracil (CMF), Fluorouracil, Adriamycin, and Cytoxan (FAC), and Fluorouracil, Epirubicin, Cyclophosphamide (FEC). The results demonstrate that higher expression of *SDC2* correlates with the resistance to Taxane (AUC = 0.581, *P* < 0.0001), Anthracycline (AUC = 0.597, *P* < 0.0001), and Ixabepilone (AUC = 0.608, *P* < 0.05). Furthermore, increased *FN* expression was associated with a better response to Taxane (AUC = 0.546, *P* < 0.01), Anthracycline (AUC = 0.574, *P* < 0.0001), and FAC (AUC = 0.591, *P* < 0.05), but with a poorer response to FEC (AUC = 0.611, *P* < 0.001), suggesting resistance to FEC-based treatments **(**Table 3**).**

**Table 3:** ROC analysis of *SDC2* and *FN* were performed across different chemotherapy treatments, comparing the gene expression of *SDC2* and *FN* between responders and non-responders breast cancer patients.

		Taxane	Anthracycline	Ixabepilone	CMF	FAC	FEC
*SDC2*	AUC:	**0.581**	**0.597**	**0.608**	0.537	0.53	0.503
	ROC *P*-value:	**1.5e-06**	**3.1e-11**	**0.033**	0.22	0.24	0.47
	responder (expression)	**487 ± 324 (n = 371)**	**473 ± 322 (n = 528)**	**360 ± 204 (n = 105)**	452 ± 341 (*n* = 53)	585 ± 288 (*n* = 62)	515 ± 362 (*n* = 84)
	Non-responder (expression)	**582 ± 389 (n = 842)**	**582 ± 389 (n = 1098)**	**464 ± 310** **(n = 31)**	540 ± 504 (*n* = 103)	665 ± 450 (*n* = 186)	535 ± 381 (*n* = 219)
	Mann-Whitney test *P*-value:	**6e-06**	**2.1e-10**	**0.07**	0.45	0.48	0.94
	Fold change:	**1.2**	**1.2**	**1.3**	1.2	1.1	1.0
*FN*	AUC:	**0.546**	**0.574**	0.588	0.511	**0.591**	**0.611**
	ROC *P*-value:	**5.3e-03**	**4.1e-07**	0.073	0.41	**0.019**	**7.6e-04**
	responder (expression)	**12,155 ± 9659 (n = 371)**	**14,186 ± 9954** **(n = 528)**	20,118 ± 8609 (*n* = 105)	12,341 ± 7743 (*n* = 53)	**11,138 ± 9931 (n = 62)**	**8884 ± 7148** **(n = 84)**
	Non-responder (expression)	**10,940 ± 9911 (n = 842)**	**11,882 ± 9937 (n = 1098)**	22,553 ± 9353 (*n* = 31)	13,403 ± 9702 (*n* = 103)	**8357 ± 9279 (n = 186)**	**12,236 ± 8804 (n = 219)**
	Mann-Whitney test *P*-value:	**0.011**	**1.3e-06**	0.14	0.83	**0.033**	**0.0028**
	Fold change:	**1.1**	**1.2**	1.1	1.1	**1.3**	**1.4**

## Discussion

In this study, we demonstrate elevated expression levels of SDC2 and FN in plasma-derived MV-enriched EVs isolated from neoadjuvant chemotherapy-naïve obese breast cancer patients with pLNM compared to those patients with nLNM.

SDCs exhibit altered expression in tumor or stromal cells, playing a crucial role in the onset and advancement of cancer via regulating signaling pathways associated with cell growth, adhesion, invasion, metastasis, cancer stem cell maintenance, and angiogenesis^32^. In addition, it has been reported that SDCs and their interaction with syntenin are essential for the biogenesis of exosomes^23^. To our knowledge, this study is the first to unravel the enrichment of SDC2 in MV-enriched EVs derived from plasma of breast cancer patients with LNM.

SDC2 appears to play a distinctive role in the progression of cancer, as a wide range of evidence indicates that it promotes tumor development^33^. It has been reported a correlation between SDC2 and enhanced migration and progression in several types of tumors, such as fibrosarcoma, melanoma and pancreatic cancer^33^. In colorectal cancer, SDC2 overexpression is strongly correlated with LNM, cancer stage, vascular invasion, and distant metastasis^34^. In normal tissue, it has been documented that SDC2 is highly expressed in mesenchymal cells, and its function appears to be intimately associated with cell migration^35^. However, in breast cancer, SDC2 has been demonstrated to be increased in the epithelial compartment, and its overexpression induces an invasive phenotype^36^. Previous studies have documented a significant upregulation of SDC2 in breast cancer cell lines relative to healthy mammary cells^37^. An investigation found that decreasing SDC2 expression in MDA-MB-231 cells resulted in a decrease in tumor size and a significant improvement in survival outcomes in a xenograft mouse model^32,38^. It has been shown that SDC2 expression is required to enhance growth and invasiveness of breast carcinoma cells by modulating cytoskeletal organization^39^.

A previous study has demonstrated that the SDC2 regulates intracellular signaling by directly interacting with FN and that interaction enhances cancer cell adhesion and dissemination^40^. In metastatic cells derived from Lewis lung carcinoma, SDC2 works with α5β1 integrin to regulate actin-cytoskeletal structure and cell adhesion to FN, which regulates their invasive capability^41^. On the other hand, it has been approved that FN was the most crucial element in promoting the upregulation of SDC2 expression among the other stromal ECM constituents, thus enhancing the migratory characteristic of highly metastatic cancer cells^42^. Indeed, our study revealed that the levels of FN derived from MV-enriched EVs were significantly higher in obese breast cancer patients with pLNM than in those with nLNM counterparts.

Among the elements of the ECM, FN has been linked to the progression of various forms of human cancer as well as cell invasion and migration in different metastatic models^43^. FN has significantly elevated expression levels in metastatic tumor locations in comparison to normal tissues and to the primary site of tumor^44^. In line with our findings, FN expression was found to be positively correlated with LNM in patients with esophageal squamous cell carcinoma (OSCC)^45^, clear cell renal cell carcinoma^46^, and thyroid carcinoma^47^. When compared to normal breast tissues, the stromal region of breast cancer exhibits much higher levels of FN protein and mRNA expression^48^. In addition, it has been demonstrated that the tumor cells in metastatic breast cancer tissues express FN at high levels^49^. Certain studies suggest that FN derived from cancer cells is associated with decreased OS and metastasis-free survival in patients with breast cancer^50^, and has a favorable correlation with LN involvement as well as Ki-67 proliferation index^51^. Research findings suggest that FN contribute to the metastasis and invasion of breast cancer by amplifying MMP-2 and MMP-9 levels^52^. It was found that FN is the primary molecule for the adherence of breast cancer cells to LN tissue^53^. EVs originating from cancer cells have been discovered to carry ECM proteins, including FN^54^. EV-derived FN improves cell attachment and internalization by activating a following signaling cascade, resulting in a variety of cellular responses^55^, and plays a role in cell motility^56^. In addition, EVs have been identified as a significant factor contributing to the accumulation of FN in the premetastatic niche^57^. It has been proposed that EV-derived FN isolated from breast cancer patients can be used for the early detection of breast cancer^58^. Collectively, these findings emphasize the significance of our results that FN derived from MV-enriched EVs of plasma of obese breast cancer patients could be a promising marker for LNM status.

The online bioinformatics tools and qPCR uncovered that *SDC2* mRNA expression was significantly reduced in breast cancer tissues with pLNM and advanced stage compared to those with nLNM and normal tissues, respectively. However, both nLNM and pLNM breast cancer patients with high expression of *SDC2* mRNA and protein levels were associated with shorter OS. Elevated FN at both the mRNA and protein levels was consistently associated with poorer OS and RFS among breast cancer patients with nLNM. This marks the clinical value of FN expression in breast cancer. The ROC curve analysis revealed that breast cancer tissue *SDC2* and *FN* individually exhibited greater diagnostic accuracy, with higher sensitivity and specificity in differentiating obese breast cancer patients with pLNM from those with nLNM. However, it is important to note that the qRT-PCR analysis was performed on a relatively small number of tissue samples. Some samples were excluded due to prior neoadjuvant treatment, non-obese status, or poor RNA quality due to Patent Blue–stained tumor tissues. Although the expression pattern was consistent with publicly available datasets supporting the reliability of our findings, the small sample size may reduce the strength and generalizability of the results. Therefore, further validation in larger and independent patient groups is needed to confirm the diagnostic value of *SDC2* and *FN* mRNA for detecting LNM. Interestingly, these findings contradict our results in MV-enriched EVs, where SDC2 and FN expression levels were notably elevated. This may suggest that during the progression of breast cancer, SDC2 and FN expressions can be shifted from tumor tissues to MV-enriched EVs.

This notion aligns with the significant inverse correlation observed for example between CD82 expression in tissues and its levels in exosomes, suggesting that exosomal CD82 content could serve as a potential indicator for assessing the metastatic capacity of cancer cells and predicting patient prognosis^59^. Together, this finding suggests the potential utility of MV-enriched EV-derived SDC2 and FN as indicators of LNM.

Additionally, several studies have reported the difference between mRNA and protein expression in various cancer types. For example, survivin expression showed inverse mRNA and protein levels in tumor tissues, which was attributed to post-transcriptional regulation mechanisms such as differential splicing, translational control, protein degradation, and intracellular localization^60^. Similarly, Audic and Hartley (2004) described how stabilization of oncogenic mRNAs in tumors can result in elevated protein levels without corresponding increases in transcript abundance^61^. In brain metastases of breast cancer, BAX protein expression was significantly higher than in primary tumors, despite reduced mRNA levels. This discrepancy was linked to post-transcriptional regulation and tumor dedifferentiation^62^. Furthermore, heterogeneity in tumor tissue samples can also contribute to such differences. The presence of stromal, vascular, immune, and necrotic cells may alter the overall transcriptomic signal, leading to variability in mRNA expression not reflected at the protein level^60^. These findings suggest that mRNA-protein discrepancies in our study may be explained by a combination of post-transcriptional control, tumor heterogeneity, and mRNA stability.

Furthermore, we conducted functional and KEGG pathway enrichment studies of SDC2 and FN. According to functional enrichment analysis, both SDC2 and FN were predominantly involved in processes associated with cell migration and protein binding. For Cellular Components (CC), both SDC2 and FN were enriched in plasma membrane and FN was enriched in extracellular exosomes and extracellular membrane-bounded organelles. KEGG pathway analysis manifested that SDC2 was mainly linked with cell adhesion and FN with ECM-receptor interaction, as well as both being associated with the proteoglycans in cancer pathway. Together, these findings suggest the significant role that could be played by SDC2- and FN-cargo-enriched MVs in metastasis.

We also used ROC Plotter and Mann-Whitney tests to thoroughly examine the response of breast cancer patients to chemotherapy with respect to *SDC2* and *FN* mRNA expression. While the results showed a trend toward differences in expression between responders and non-responders, the predictive accuracy was modest, with AUC values around 0.57–0.60. These findings suggest that *SDC2* and *FN* mRNA may act as promising predictive biomarkers for chemotherapy response when combined with other biomarkers or clinical features. Further research is needed to confirm their significant role in predicting chemotherapy outcomes in breast cancer.

One of the key strengths of our study lies in the use of a rapid, straightforward, and cost-effective isolation protocol for MV-enriched EVs, which was achieved using differential centrifugation, avoiding the need for ultracentrifugation or commercial isolation kits. This method offers a practical alternative suitable for large-scale or resource-limited research. However, a potential limitation associated with this study is the low yield of proteins derived from MV-enriched EVs. Therefore, we overcame this limitation by pooling EV samples. While pooling allowed for detection of EV protein cargo, it limits the ability to capture inter-individual variability and reduces the statistical power of our comparisons. Statistical analyses were therefore based on the number of pools rather than individual patient samples. Future studies using single-patient-derived EV samples will be essential to validate and extend these findings. Furthermore, we acknowledge the need for future studies to examine these findings in non-obese breast cancer patients to enhance their generalizability and clinical relevance.

## Conclusion

In conclusion, our study is the first to underscore that MV-enriched EVs isolated from neoadjuvant chemotherapy-naïve obese breast cancer patients contained SDC2 and FN, serving as a potential non-invasive biomarker for detection of LNM, further, breast cancer tissue-derived *SDC2* and *FN* mRNA levels act as diagnostic markers to differentiate breast cancer patients with pLNM from those with nLNM. Future research is essential to unravel the molecular mechanisms driving the elevated levels of SDC2 and FN in MV-enriched EVs and their role in preparing the metastatic niche for breast cancer cells.

## Materials and methods

### Clinical sample collection

This study was approved by the institutional review board (IRB00012829) of Baheya-Research Ethics Committee, Giza, Egypt. Venous blood samples from women diagnosed with primary breast cancer were obtained prior to modified radical mastectomy or breast conservative curative surgery and all patients signed an informed consent to participate in the study. All methods were carried out in accordance with relevant regulations and with the Declaration of Helsinki. The inclusion criteria were as follows: Adult female patients aged ≥ 18 years, obese with BMI > 30, and pathologically confirmed primary breast cancer, stage I-III (non-metastatic). Patients were excluded if diagnosed with stage IV, as well as those with serious conditions like autoimmune disorders or received neoadjuvant treatment. Additionally, any samples that were hemolyzed or clotted after collection were excluded. Among the 104 samples obtained from breast cancer patients, only 39 patients met the specified inclusion and exclusion criteria. The number of non-obese breast cancer patients was limited and did not meet the inclusion criteria—specifically, having undergone neoadjuvant chemotherapy—thus precluding statistically powered comparisons. Therefore, we focused our analyses on the neoadjuvant chemotherapy-naïve obese breast cancer to ensure statistical robustness and clinical relevance. Blood samples were collected in Acid-Citric-Dextrose (ACD) tubes and platelet-poor plasma (PPP) was separated by centrifugation twice at 2500 × g for 15 min at room temperature (RT)^63^.

### Histopathology and immunohistochemistry (IHC)

Breast cancer tissues were fixed in 4% neutral-buffered formalin solution, underwent standard processing and were then embedded in paraffin. The initial diagnosis was based on H&E-stained tissue sections. The IHC assay was conducted on 5-μm slices, which were subjected to de-waxing in xylene and rehydration in a declining series of ethyl alcohol as described before^64^. The process of antigen retrieval was achieved by subjecting the samples to heat treatment in a steamer, using a citrate buffer (pH 6.1) and hydrogen peroxide was used to suppress endogenous peroxidase activity. Subsequently, sections were exposed to primary antibodies targeting ER, PR, HER2, and Ki-67 for 1 h at RT. Finally, chromogen solution (DAB + and substrate buffer) was used for immunodetection, and hematoxylin was used for counterstaining.

In order to determine the HER2 gene status for the tissue samples that were equivocal, we followed the previously published procedure using silver in situ hybridization (SISH)^65^. Positive HER2 result is indicated by amplified HER2 staining, while negative HER2 is indicated by non-amplified HER2 staining. The ER, PR, HER2 and SISH data were reported in accordance with the latest guidelines from the American Society of Clinical Oncology (ASCO)/College of American Pathologists (CAP) in 2018^66^.

### Isolation of EVs

For isolation of EVs, the PPP was centrifuged for 1 h at 21,000 × g at 4 °C. The pellet was resuspended in 0.22 μm pre-filtered phosphate buffered saline (PBS) re-centrifuged twice for 1 h at 21,000 × g at 4 °C. Finally, the EV pellet was lysed in RIPA buffer (50mM Tris-HCL (pH 7.4), 150 mM NaCl, 1% (v/v) Triton X-100, 1mM EDTA, 1% (v/v) Sodium deoxycholate, 0.1% SDS) containing protease inhibitor cocktail with sonication for 20 min as we described before^67^. The BCA assay kit (Serva, Heidelberg, Germany) was used to measure the protein content of the isolated EVs in nanoquant Tecan Infinite PRO 200 (Tecan, Switzerland) following the manufacturer’s instructions.

### Confirmation and characterization of isolated EVs

We used DLS, TEM, dot blot and western blot to confirm the successful isolation of EVs as we previously described^67^.

### Dynamic light scattering (DLS)

The size and PDI of the isolated EVs were examined via Zeta sizer nano series (ZEN 3600, Malvern, UK) at the core facility of Nanotechnology and Advanced Materials Central Lab, Agricultural Research Center. The pellet of EVs was dissolved in 3 mL 0.22 μm pre-filtered PBS and mixed well to obtain homogenous solution. The analysis was performed in triplicates at 25 °C with a count rate of 157.8 kcps, measurement position 3 mm and duration 70 s.

### Transmission electron microscopy (TEM)

We used high-resolution TEM (HR-TEM) to determine the morphology and size of the isolated EVs. A few drops of EVs suspension in PBS were applied on carbon coated copper grid and left to dry. Next, a few drops of phosphotungstic acid (1%) were added on the grid and left to dry. Finally, the grid was examined by HR-TEM (JEOL, JEM-2100, Tokyo, Japan) at 200 kv.

### Dot blot and Western blot

Equal protein concentrations (5 µg) from the isolated MV-enriched EVs were diluted in 3× reducing loading buffer (1 M Tris- HCl (pH 6.8), 30% glycerol, 6% SDS, 3% 2-mercaptoethanol, 0.005% bromophenol blue) and the mixture was heated at 95 °C for 5 min. Afterward, the samples were loaded onto a 7.5%, or 12% SDS-PAGE and semi-dry electro-transferred onto a nitrocellulose membrane (Amersham, UK). The membranes were blocked with 5% skimmed milk (Serva, Heidelberg, Germany) in TBST buffer (10 mM Tris, 150 mM NaCl, 0.1% Tween 20) for 1 h at RT, followed by incubation overnight at 4 °C with primary antibodies diluted at 1:1000 CD9 (Invitrogen (Ts9)), HSP70/HSC70 (Santa-Cruz (W27): sc-24), Calnexin (Santa-Cruz (H-70): sc-11397), Syndecan-2 (Santa-Cruz (M-140): sc-15348) and Fibronectin (Santa-Cruz (EP5): sc-8422). After washing two times for 5 min and one time for 20 min with TBST, the membranes were incubated with horseradish peroxidase (HRP)-labelled goat anti‐mouse (Gaithersburg, Maryland, USA) or goat anti‐rabbit secondary (Merck-Millipore, Billerica, MA, USA) antibodies diluted at 1:2000 for 1 h at RT. Finally, we added chemiluminescent HRP substrate for development of the signal after washing with TBST thrice for 5 min each time. We used the UVP Biospectrum Imaging System and Image Acquisition (Analytik Jena, Cambridge, UK) for signal visualization. The loading control for MV-enriched EVs employed in the western blot experiments was CD9 and the blots were prepared in parallel under non-reducing conditions. Due to the limited protein yield from individual MV-enriched EV samples, western blot analyses were performed using pooled lysates from individual patient samples for each group (nLNM and pLNM). Consequently, statistical comparisons were conducted using the number of pooled samples (*n* = 3 per group).

Dot blots were carried out by spotting 2 µL of the protein lysate samples with equal concentration onto a nitrocellulose membrane and then were left to dry in air. After blocking the membrane for 1 h, all the next steps were analogously repeated as mentioned in western blot. Details of the membrane’s cuts and their respective exposure durations are available in **Supplementary Material 1**.

### Protein-protein interaction (PPI) network of SDC2

The STRING database v.11 (http://string-db.org/) (accessed on 25 August 2024) is a comprehensive and objective worldwide network that gathers, combines, and scores all documented PPI data^68^. It further enhances this data with scientific calculations and predictions to provide entire physical and functional PPI networks. We constructed a PPI network linked to SDC2 using the STRING database.

### ELISA

The concentration of FN in plasma-derived MV-enriched EVs isolated from both nLNM and pLNM breast cancer patients were quantified using an ELISA kit (Wuhan Elabscience Biotechnology, Wuhan, China) following the instructions provided by the manufacturer. Equal protein concentration of MV-enriched EV lysates was used. Following development using a chromogen-substrate solution, 100 µL of stop solution was added. Optical density readings were measured at 450 nm, and concentrations were computed automatically using the standard curve and multiplied by the dilution factor.

### Enrichment analysis of SDCs and FN

The DAVID database (https://david.ncifcrf.gov/) (accessed on 25 August 2024) is a powerful bioinformatics tool to annotate and assess the biological functions of genes and proteins, providing valuable insights into their roles and interactions^69^. We used DAVID and STRING databases to conduct functional enrichment analysis according to biological process (BP), cellular component (CC), molecular function (MF), subcellular localization and KEGG pathway^70^. Statistical significance was determined for *P*-values less than 0.05.

### Total RNA extraction and quantitative Real-Time PCR

Total RNA extraction from fresh tumor tissue samples was performed using QIAzol lysis reagent (Qiagen, Hilden, Germany) and the GeneJET RNA Purification Kit (Thermo Fisher Scientific, USA), following the protocol provided by the manufacturer. After quantification of RNA concentration and purity using Infinite^®^200 PRO NanoQuant (Tecan, Switzerland), 1 µg of RNA was reverse transcribed into cDNA using the High-Capacity cDNA Reverse Transcription Kit (Thermo Fisher Scientific, CA, USA), in accordance with the manufacturer’s protocol. Next, the SYBR™ Green PCR Master Mix (Applied Biosystems, USA) was used to analyze the expression levels of *SDC2* and *FN* in breast carcinoma tissues from 9 nLNM and 13 pLNM patients using StepOnePlus Real-Time PCR System (Applied Biosystems, CA, USA). The relative expression of genes was assessed through the fold-change calculation method 2^−∆∆Ct^, as previously described^71^ and data were normalized using *18S* rRNA gene as housekeeping gene for its stability under the experimental conditions^72^. Primers are listed in Supplementary Table 1.

### Analysis of SDC2 and FN expression in breast cancer

UALCAN (http://ualcan.path.uab.edu/) (accessed on 25 August 2024) is a publicly accessible resource for cancer information analysis, providing transcriptional expression database based on the TCGA data and protein profiling using Clinical Proteomic Tumor Analysis Consortium (CPTAC) dataset^73^. We evaluated the mRNA and protein levels of SDC2 and FN in breast cancer and corresponding normal tissues based on TCGA and CPTAC data in UALCAN. Additionally, the HPA database (https://www.proteinatlas.org/) (accessed on 25 August 2024) was used to validate the protein level of SDC2 and FN in both normal and cancer tissues through IHC analysis^74^.

### Clinicopathological value of *SDC2* and *FN* expression in breast cancer

To investigate the associations between the expression of *SDC2* and *FN* and various breast cancer characteristics in the TCGA dataset, we utilized UALCAN and cBioPortal (https://www.cbioportal.org/) (accessed on 25 August 2024)^75^ web tools. We focus on two main characteristics of breast cancer, including nodal metastasis status and cancer stages. We conducted a comparative analysis of *SDC2* and *FN* expressions between several breast cancer subgroups and normal.

### Survival analysis of SDC2 and FN in breast cancer

The Kaplan–Meier Plotter (http://kmplot.com/analysis/) (accessed on 25 August 2024), an online tool containing gene expression data and survival statistics of different types of cancer^76^, was utilized to assess the prognostic value of *SDC2* and *FN* mRNA and protein expression in association with OS and RFS in the whole patients of breast cancer or classified according to LN status. Patients were classified as having low or high SDC2 and FN expression based on the median value.

### ROC Plotter for treatment response evaluation

The ROC Plotter platform (http://www.rocplot.org/) (accessed on 24 October 2024) was utilized to examine the association between *SDC2* and *FN* gene expression and pathological complete response to various treatments, based on transcriptomic data from breast cancer patients^77^. The breast cancer samples in the dataset were divided into two groups, responders and non-responders, based on their clinical profiles. These groups were then analyzed with Mann-Whitney U and ROC tests. Statistical significance was defined by a *P*-value threshold of *P* < 0.05, and only results having a false discovery rate (FDR) of 5% or lower were deemed significant.

It should be noted that the online datasets (STRING, DAVID, UALCAN, HPA, cBioPortal, Kaplan–Meier Plotter, and ROC Plotter) do not allow filtering of the selection of breast cancer patients based on BMI.

### Statistical analysis

Data are presented as mean and standard deviation (SD) or standard error mean (SEM) as indicated. A normality test was performed using skewness and kurtosis, while parametric tests were utilized to analyze data that was normally distributed. To analyze and compare two groups of data that followed a normal distribution, we employed the unpaired Student’s t-test. On the other hand, data from multiple groups were analyzed using one-way ANOVA, which was then followed by the Tukey post-HOC test. For non-parametric data, statistical significance was evaluated using the Chi-square test. SPSS software (version 27) was used to analyze the data, and GraphPad Prism 8 was used for elaborating the graphs. Results were deemed significant if the *P*-value was less than 0.05 for all tests.

## Supplementary Information

Below is the link to the electronic supplementary material.

Supplementary Material 1

## Data Availability

The datasets generated during and/or analysed during the current study are available from the corresponding author upon reasonable request.
