# Supplementary material for: SDC2 and FN as cargo proteins in circulating extracellular vesicles in obese breast cancer patients with lymph node metastasis
Source: Sci Rep. 2025 Sep 12;15:32498. doi: 10.1038/s41598-025-17638-2 (PMC12432118; doi:10.1038/s41598-025-17638-2)
Supplement: Supplementary file 1 — Supplementary Material 1 [file 41598_2025_17638_MOESM1_ESM.pdf]

Table S1. List of primers used in this study.

Gene		Sequence
SDC2	F	ACTGTTGACTAGTGCTGCTCCA
	R	GGGTCCATTTTCCTTTCTGAGT
FN1	F	ACAACACCGAGGTGACTGAGAC
	R	GGACACAACGATGCTTCCTGAG
18S	F	AACCCGTTGAACCCCATT
	R	CCATCCAATCGGTAGCG

Supplementary Fig.S1a. Dot blot for CD9

1 min exposure time

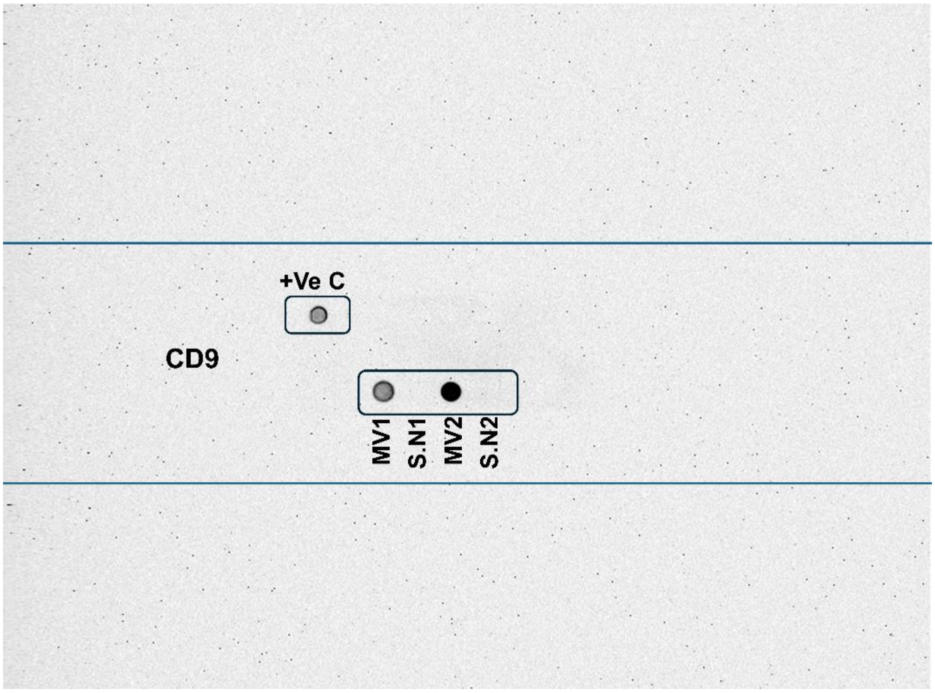

5 min exposure time

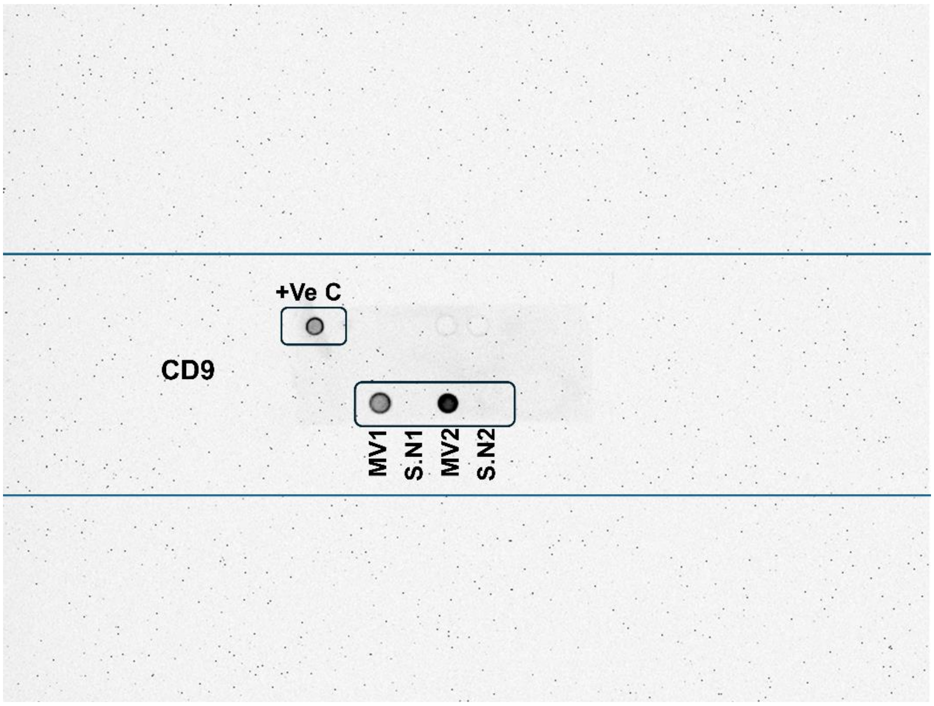

30 min exposure time

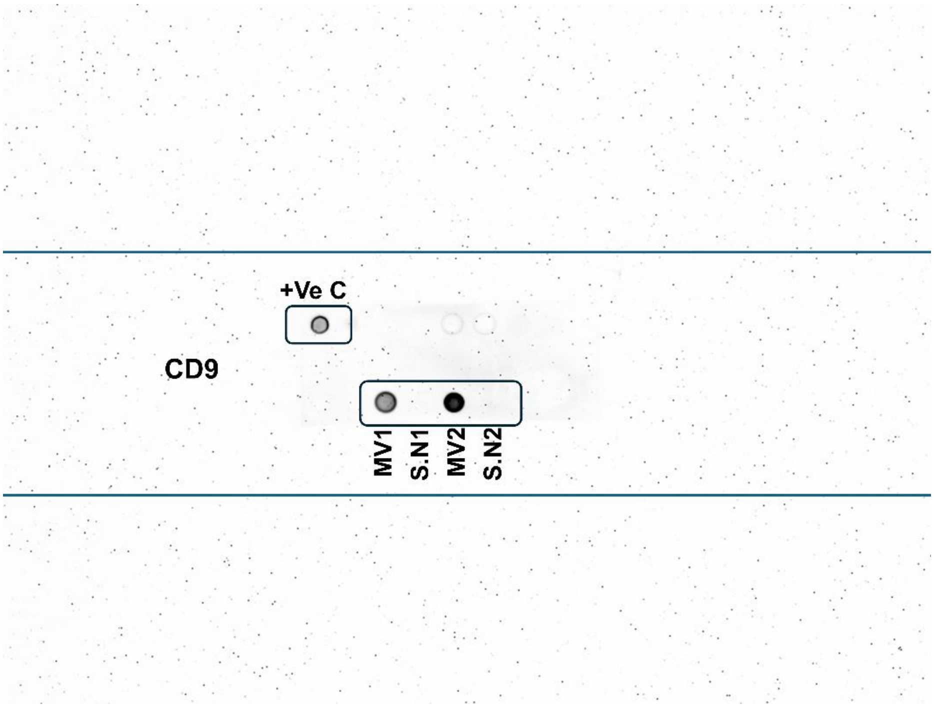

Supplementary Fig. S1a. Raw data for fig. 2C. Dot blot analysis for MV marker CD9 using varying exposure durations.

Supplementary Fig. S1b. Dot blot for HSP70

10 min exposure time

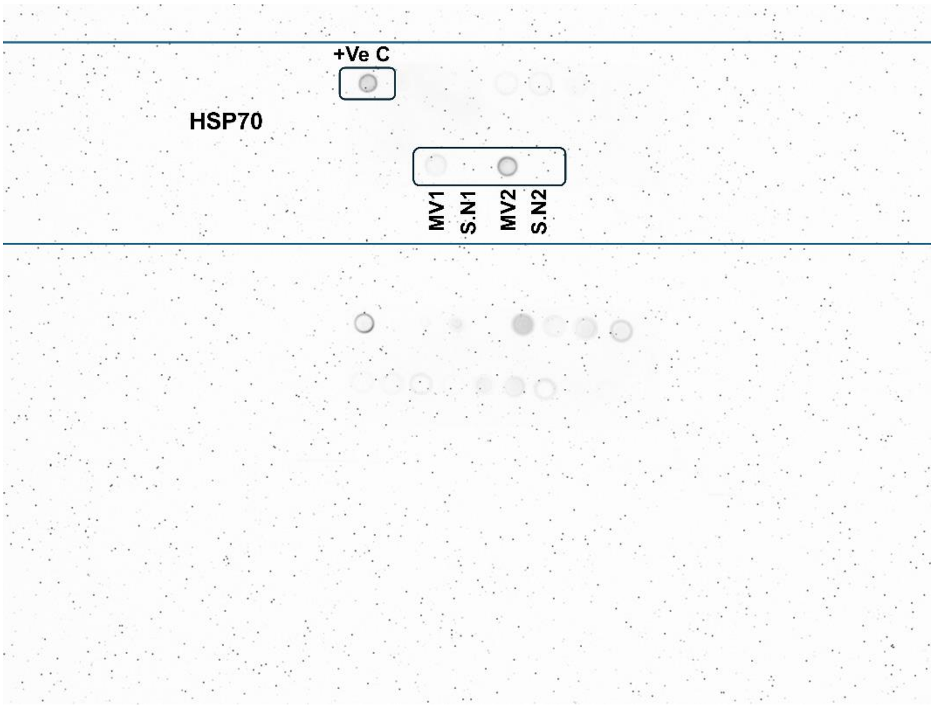

30 min exposure time

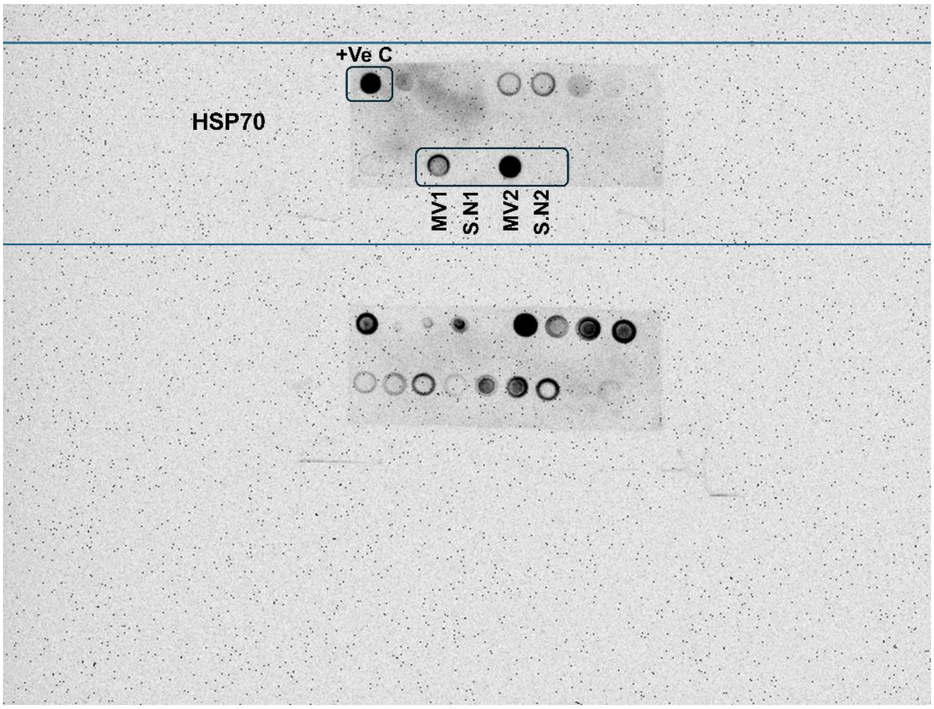

Supplementary Fig. S1b. Raw data for fig. 2C. Dot blot analysis for MV marker HSP70 using varying exposure durations.

Supplementary Fig. S1c. Western blot of CD9

Membrane Cut

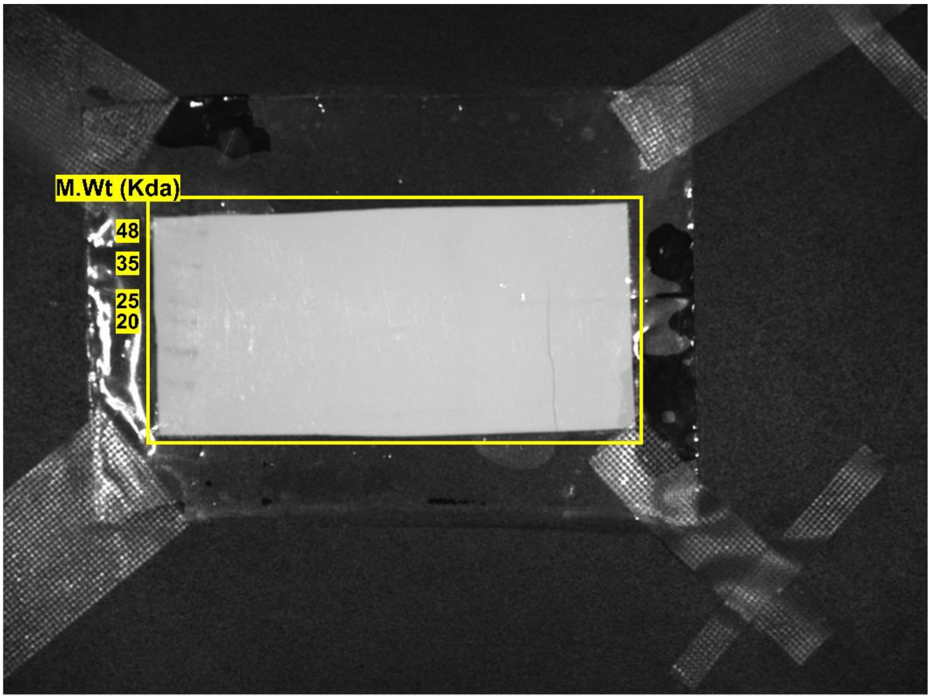

5 min exposure time

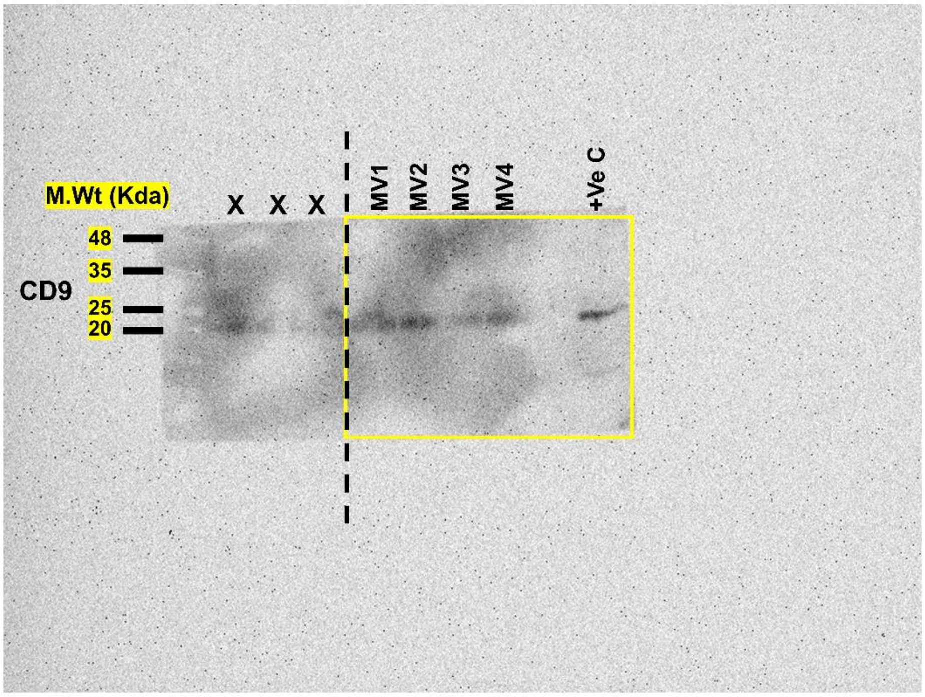

20 min exposure time

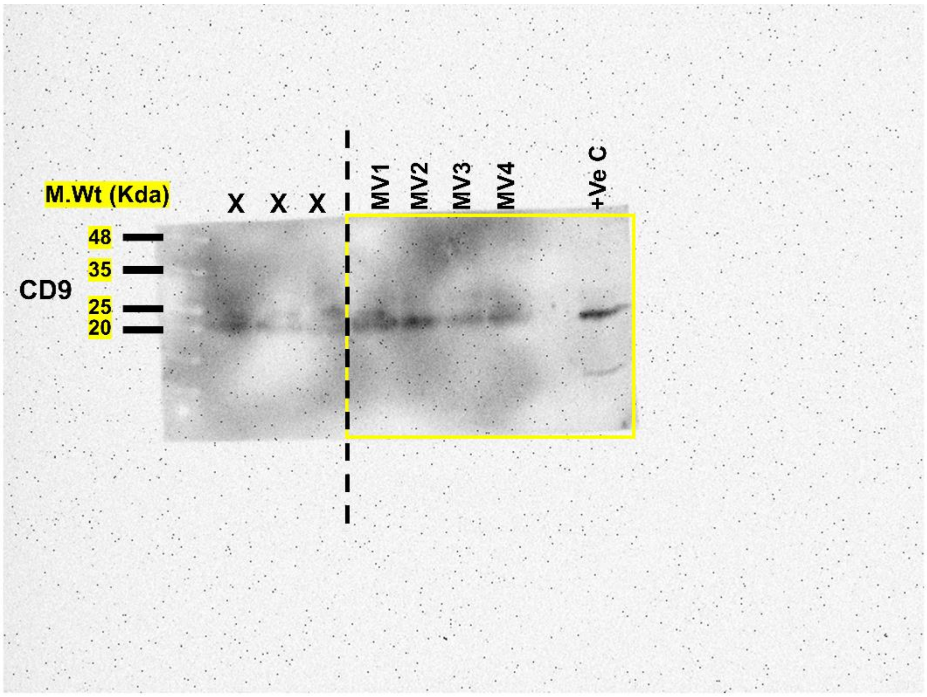

30 min exposure time

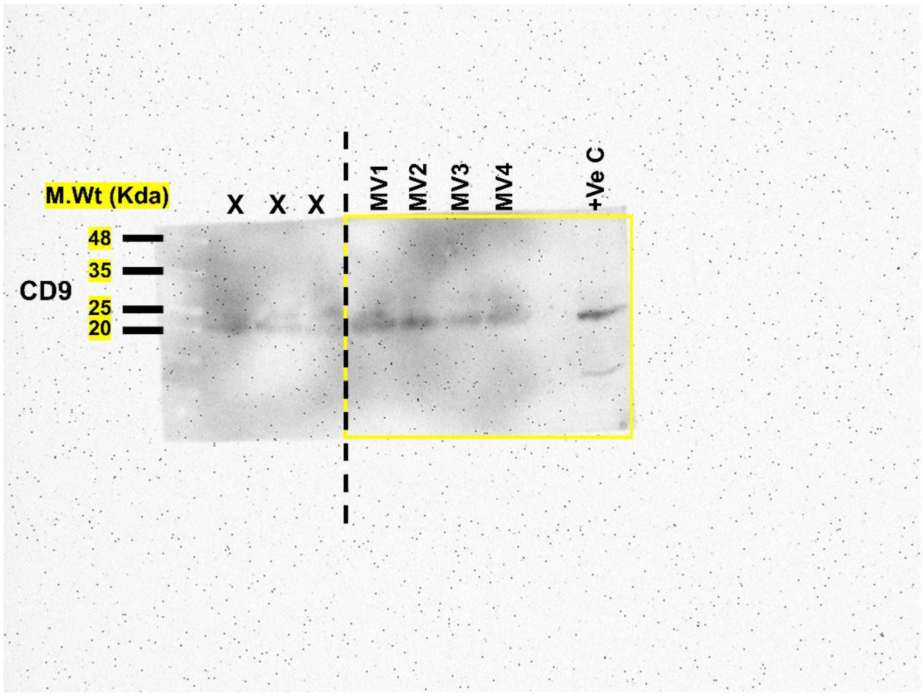

Supplementary Fig. S1c. Raw data for fig. 2D. Western blot analysis of CD9 in MV samples. Images for membrane cut and blot with varying exposure durations.

Supplementary Fig. S1d. Western blot of HSP70

Full membrane Ponceau S stain

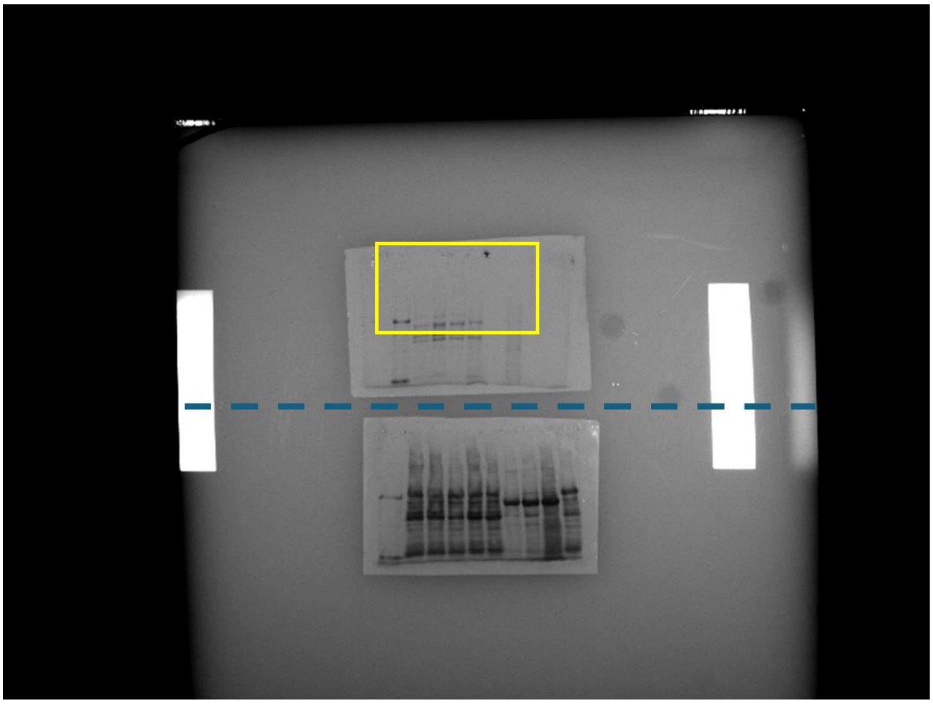

Membrane Cut

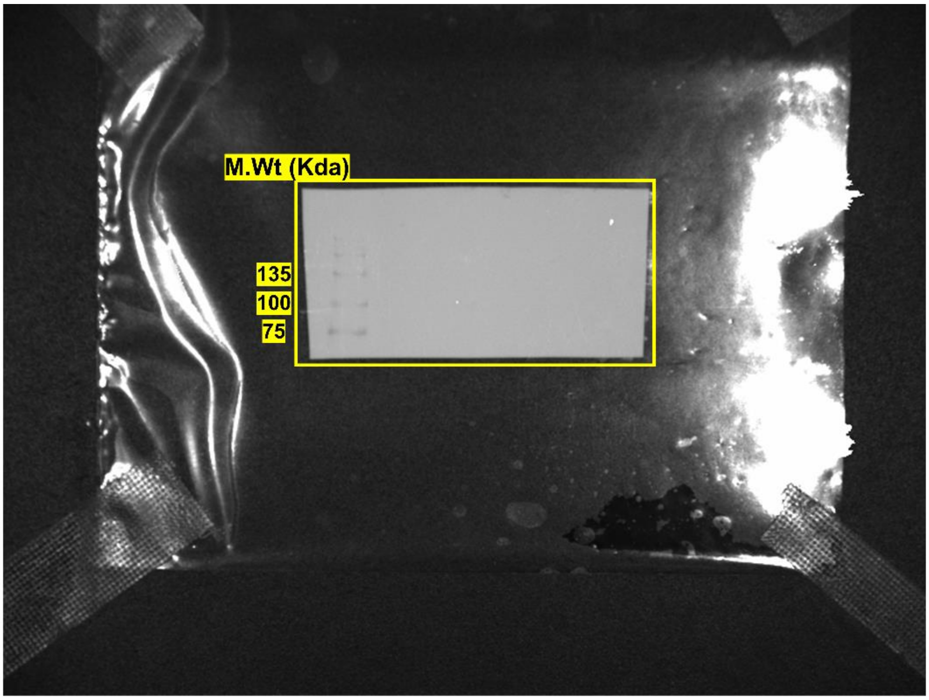

5 min exposure time

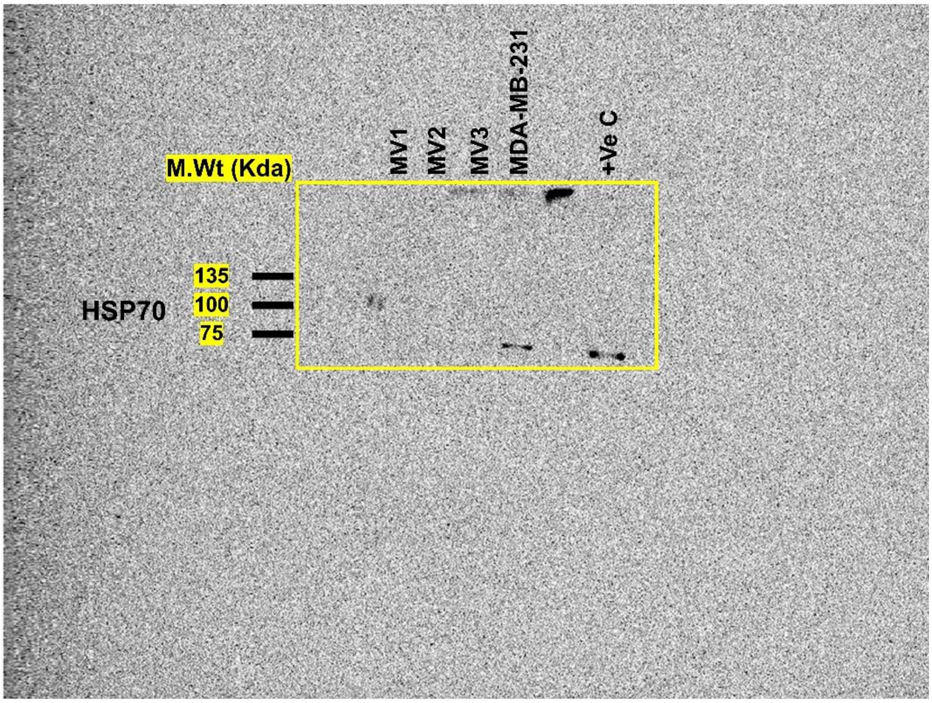

30 min exposure time

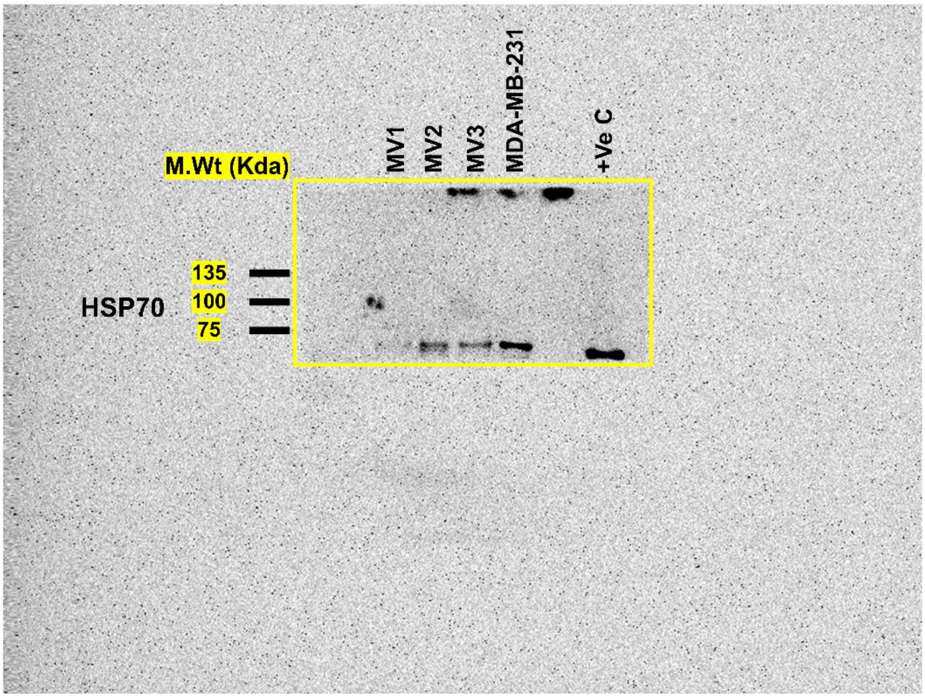

40 min exposure time

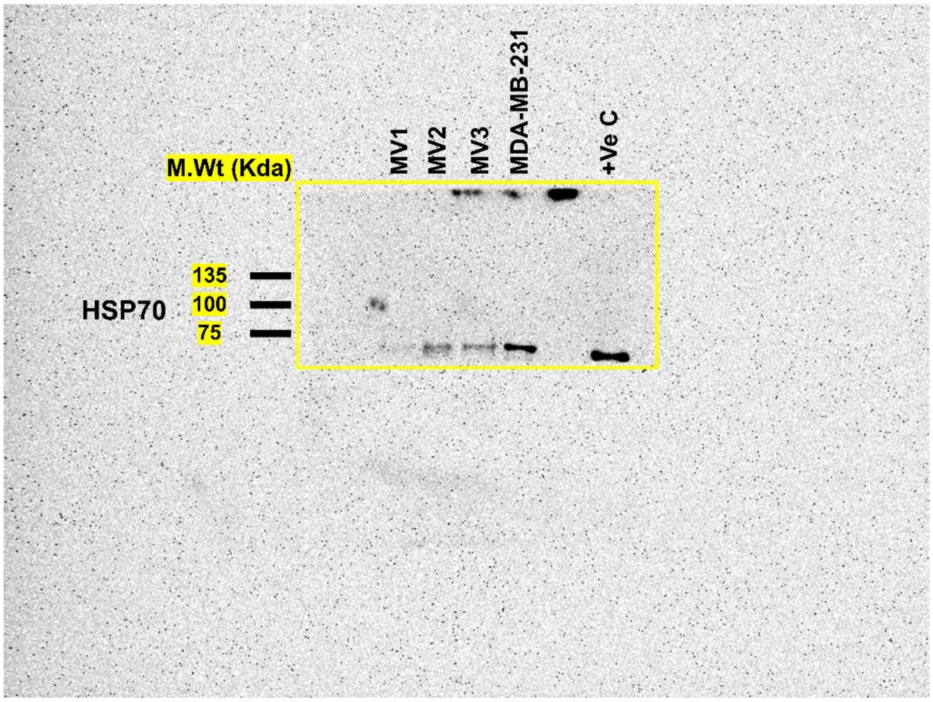

Supplementary Fig. S1d. Raw data for fig. 2D. Western blot analysis of HSP70. Images for Ponceau S staining, membrane cut and blot with varying exposure durations.

Supplementary Fig. S1e. Western blot of Calnexin

Full membrane Ponceau S stain

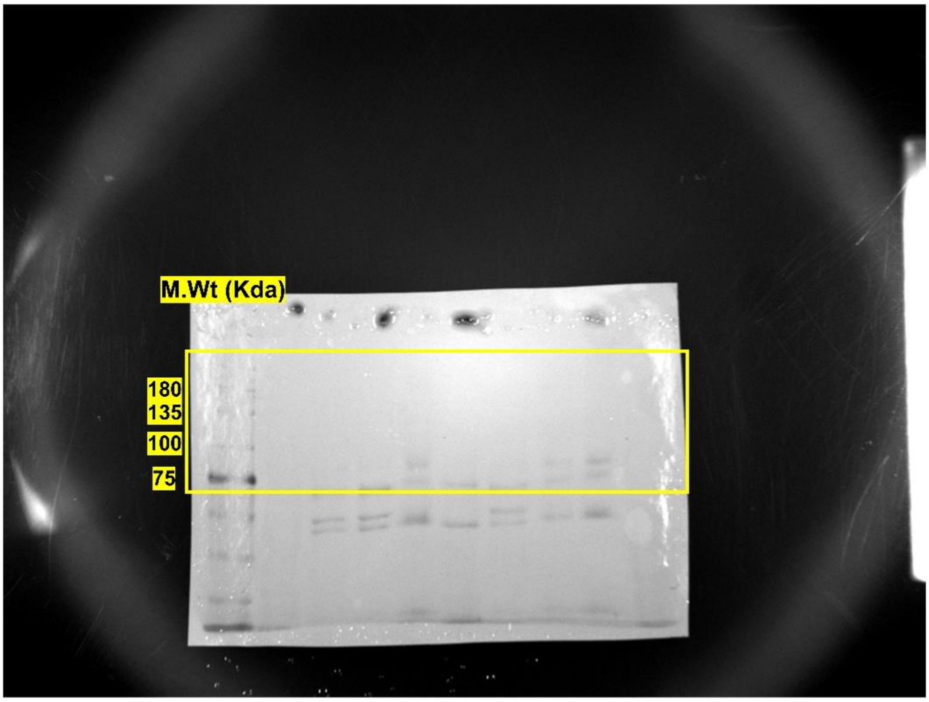

Membrane Cut

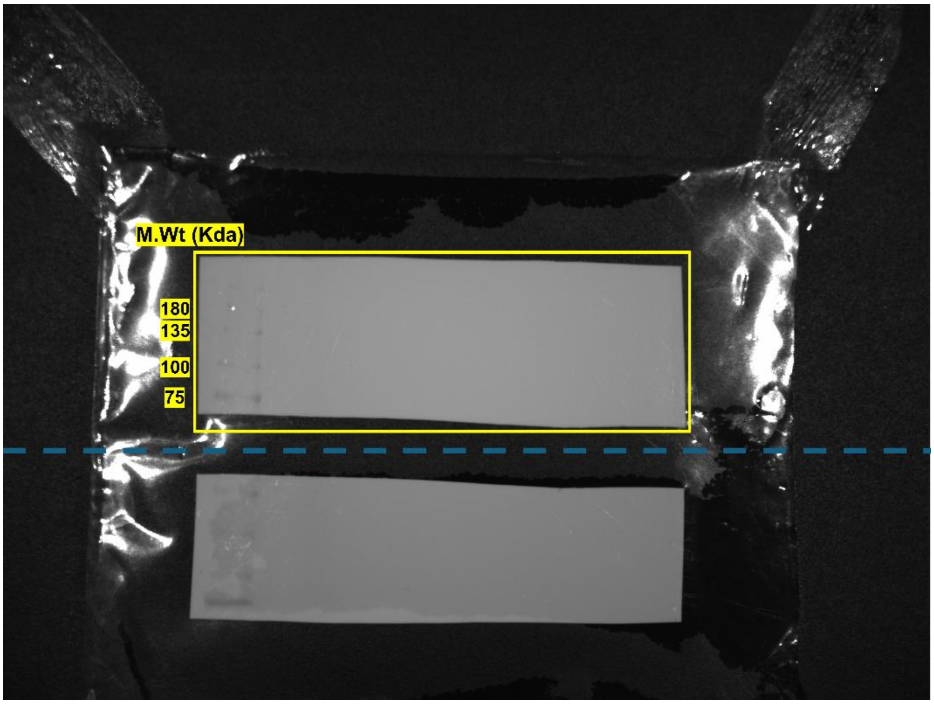

5 min exposure time

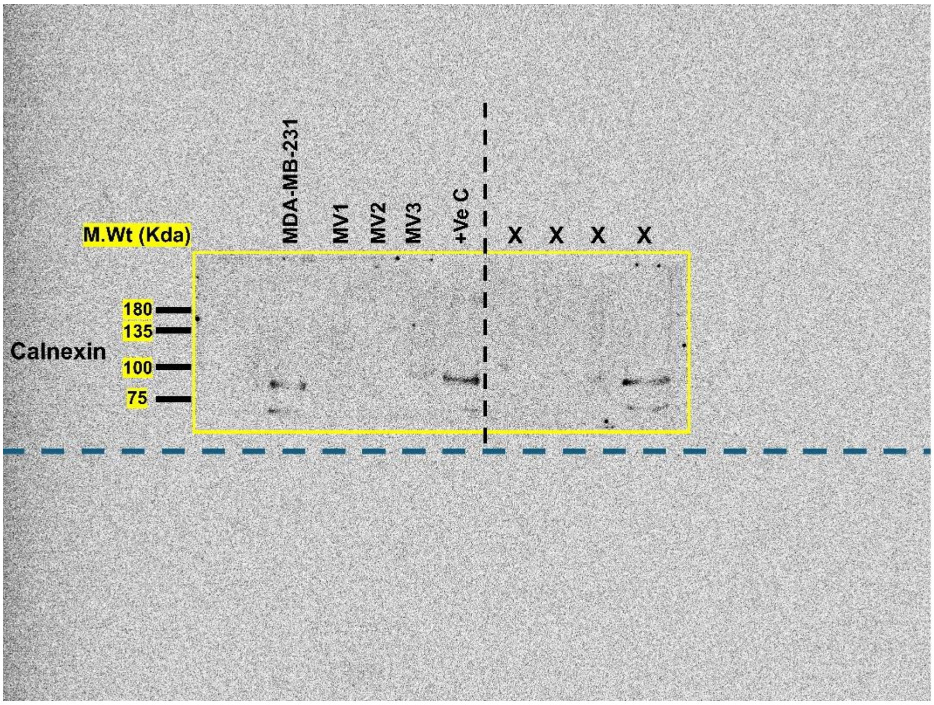

10 min exposure time

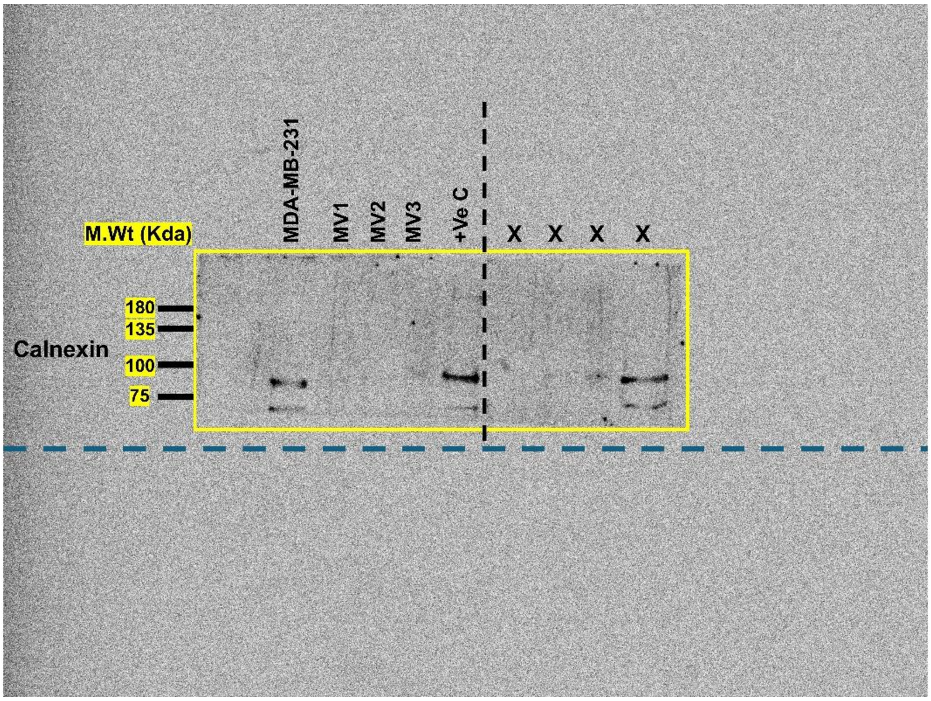

30 min exposure time

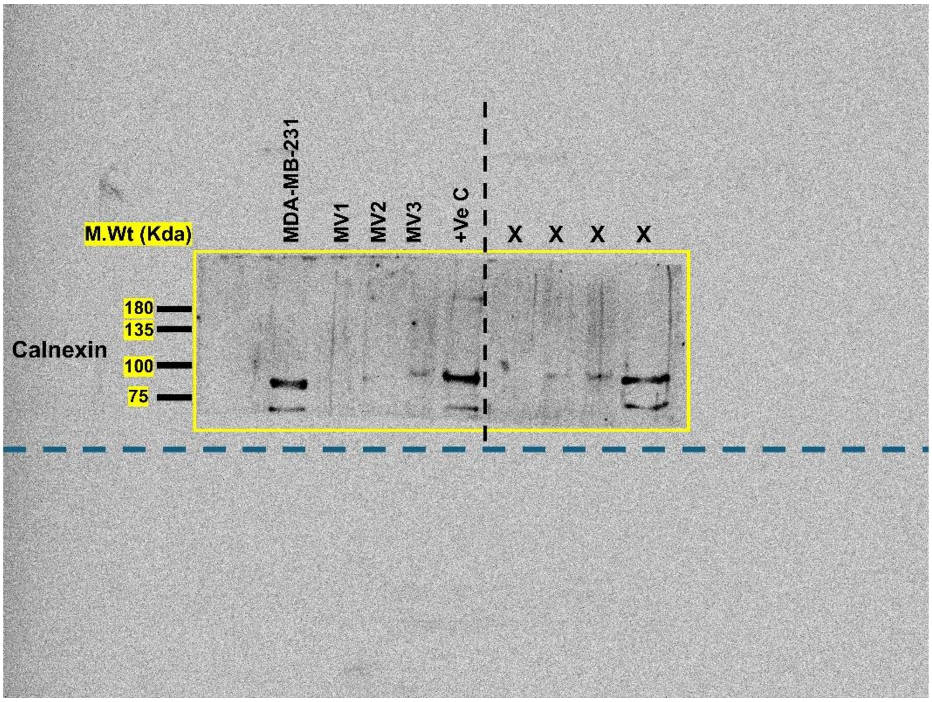

Supplementary Fig. S1e. Raw data for fig. 2D. Western blot analysis of Calnexin. Images for Ponceau S staining, membrane cut and blot with varying exposure durations.

Supplementary Fig. S2a. Dot blot for SDC1, SDC2 and SDC4

Membrane Cut

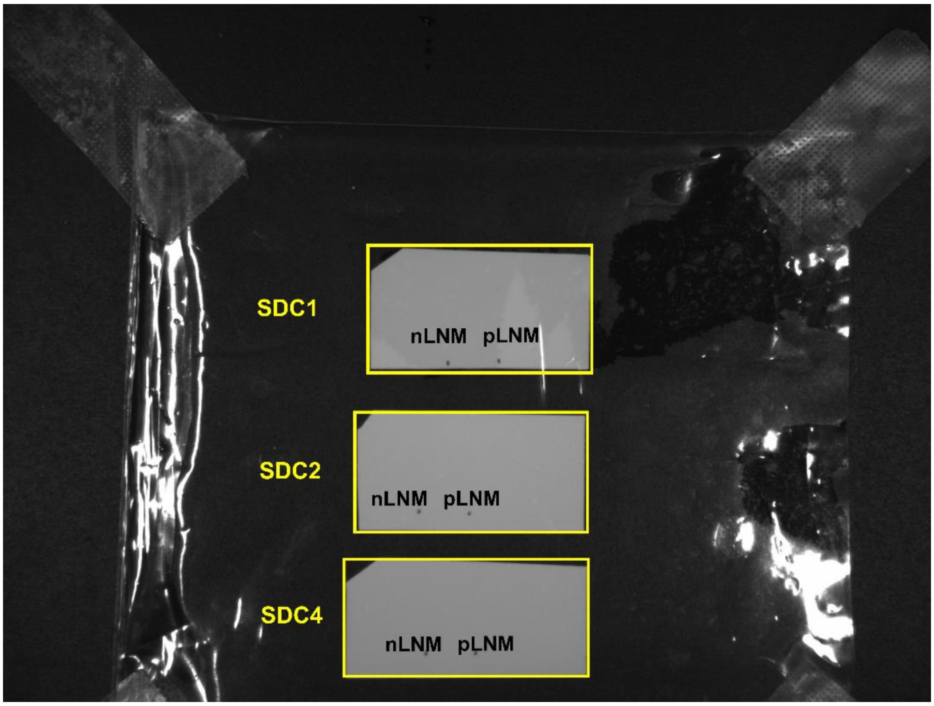

1 min exposure time

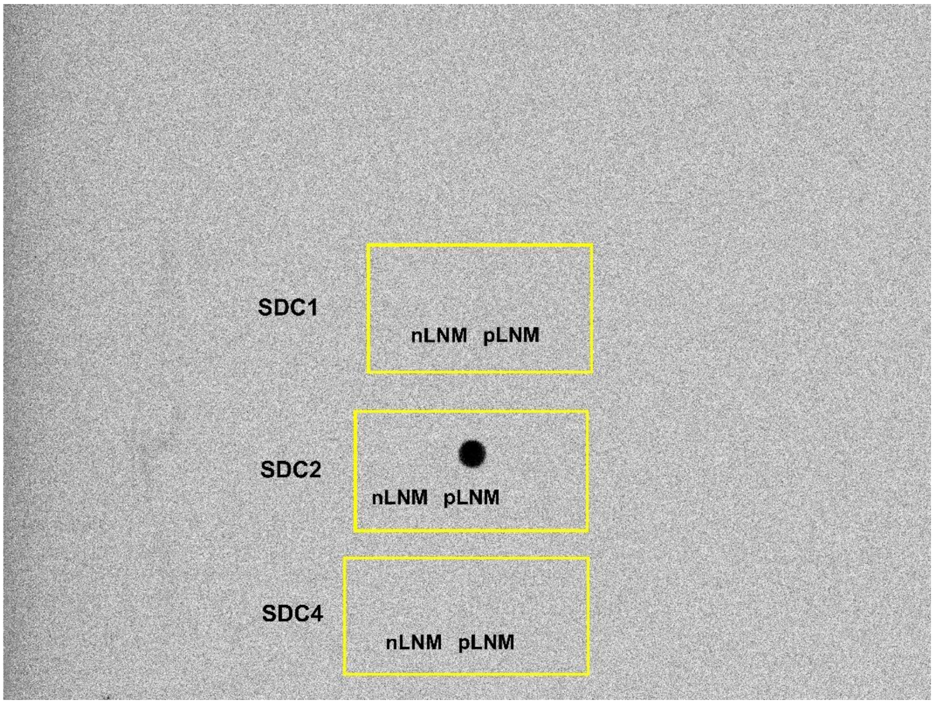

5 min exposure time

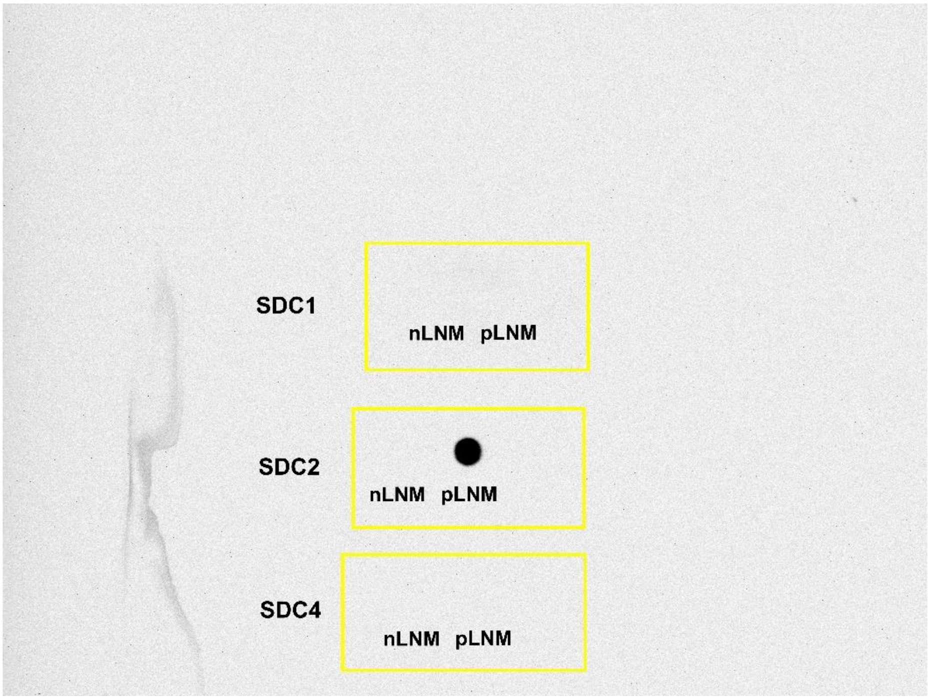

30 min exposure time

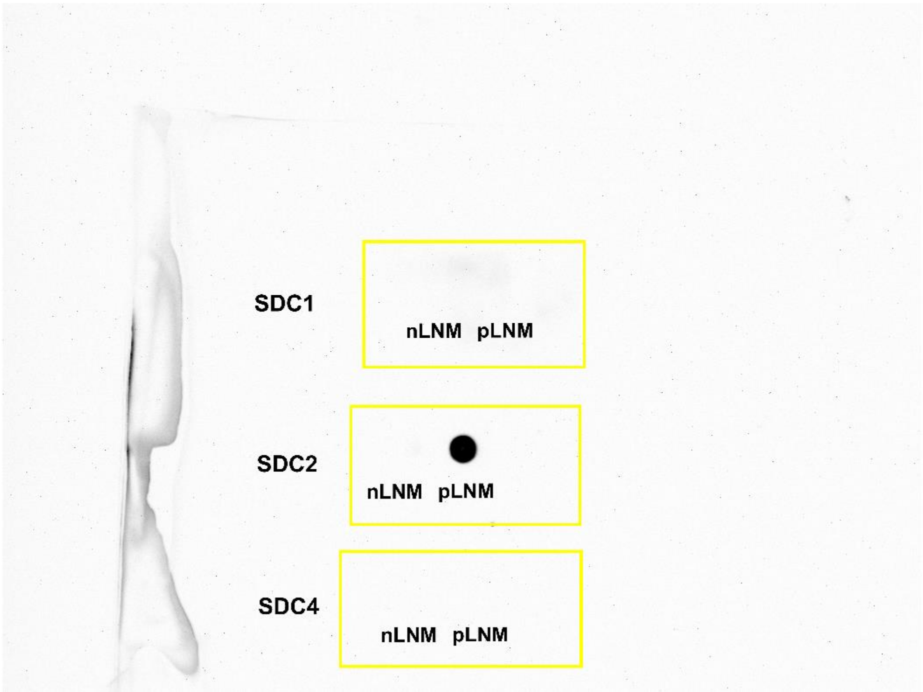

Supplementary Fig. S2a. Raw data for fig. 3A. Dot blot analysis for SDC1,SDC2 and SDC4. Images for membrane cut and blot with varying exposure durations.

Supplementary Fig. S2b. Western blot of SDC2

Replicate 1

Full membrane Ponceau S stain

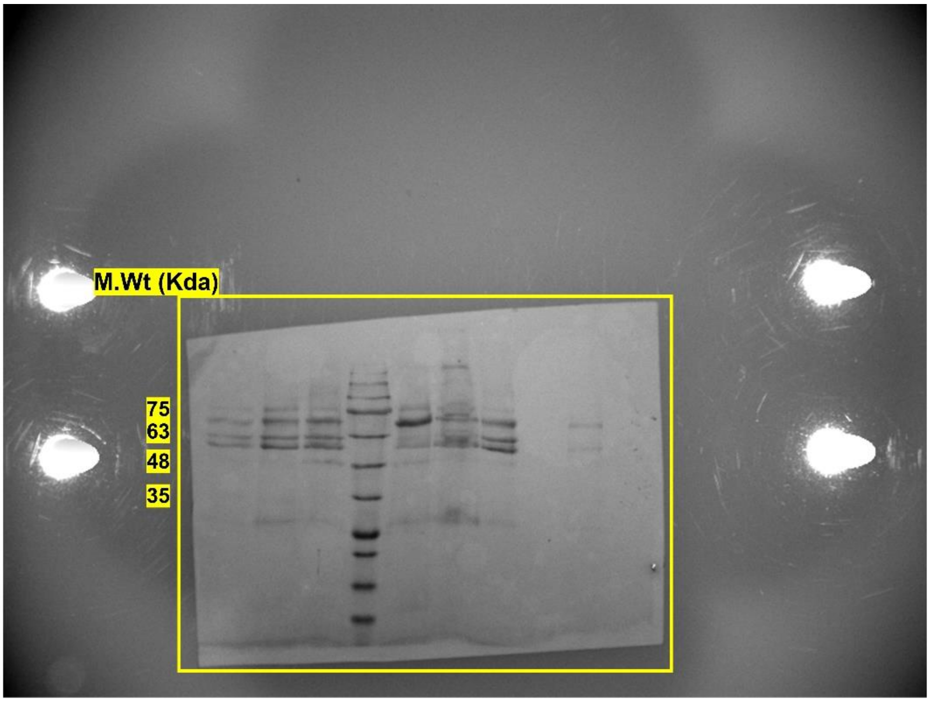

Membrane Cut

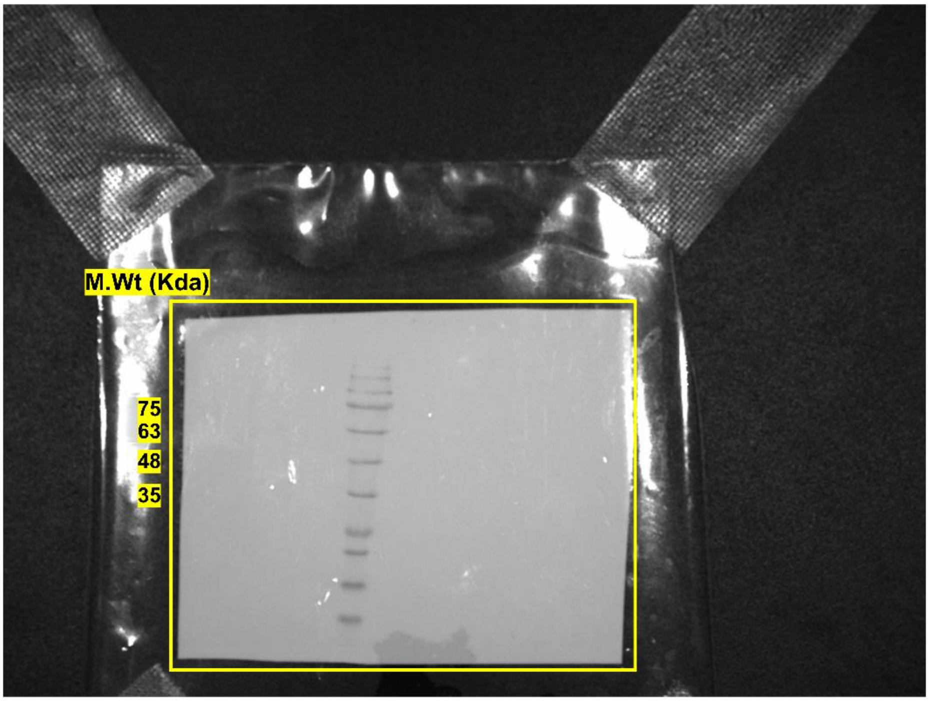

5 min exposure time

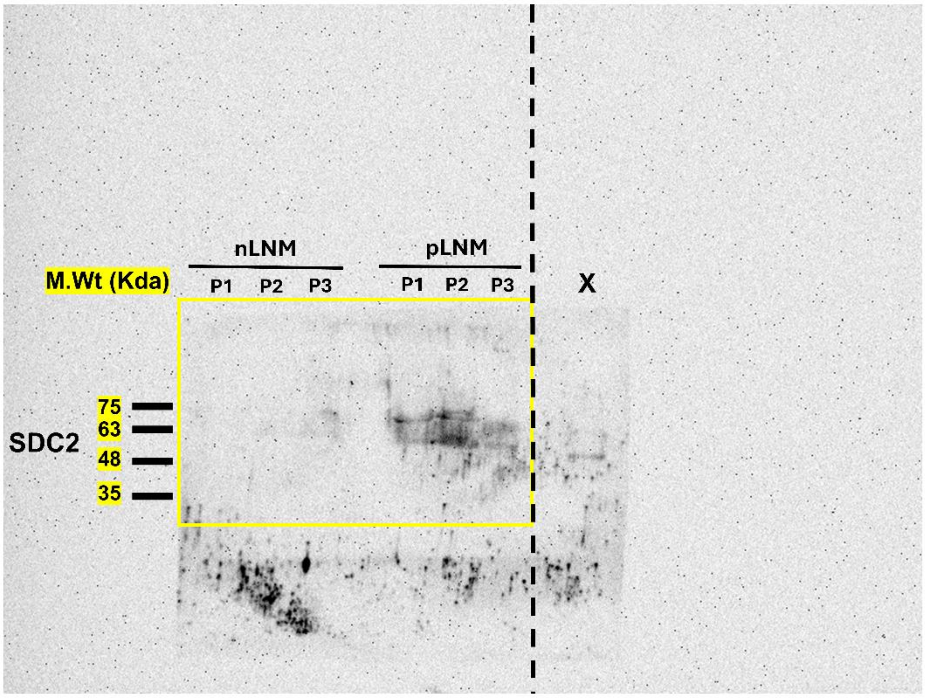

10 min exposure time

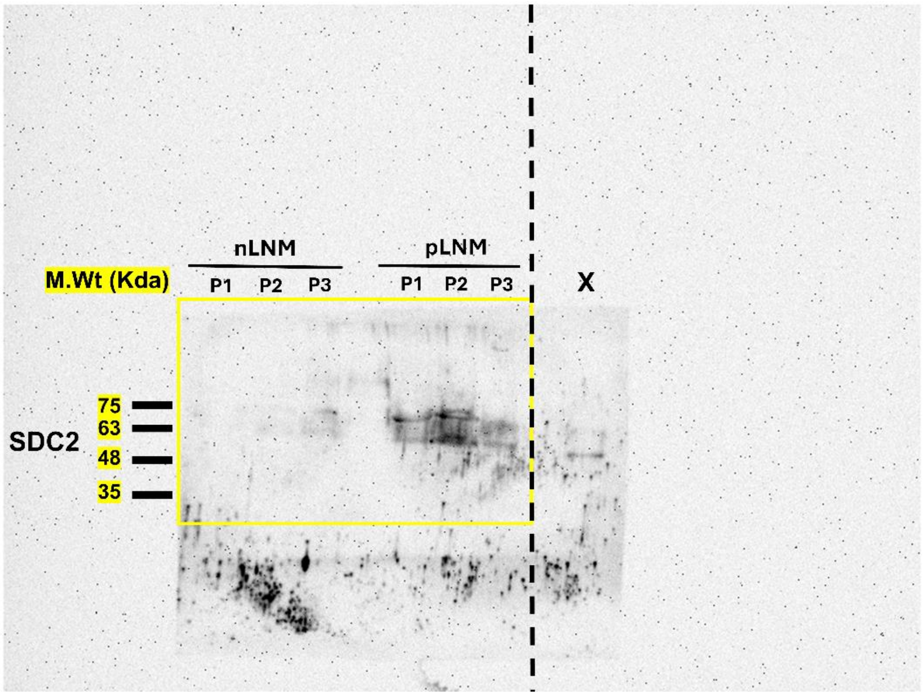

20 min exposure time

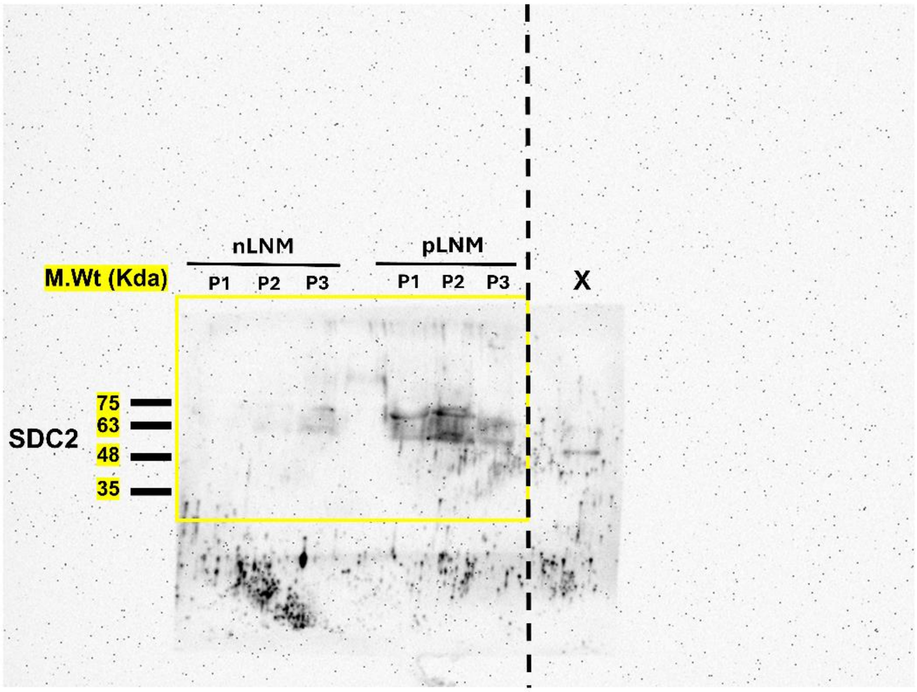

Supplementary Fig. S2b. Raw data for fig. 3B. Western blot analysis of SDC2. Images for Ponceau S staining, membrane cut and blot with varying exposure durations.

Supplementary Fig. S2b. Western blot of SDC2

Replicate 2

Full membrane Ponceau S stain

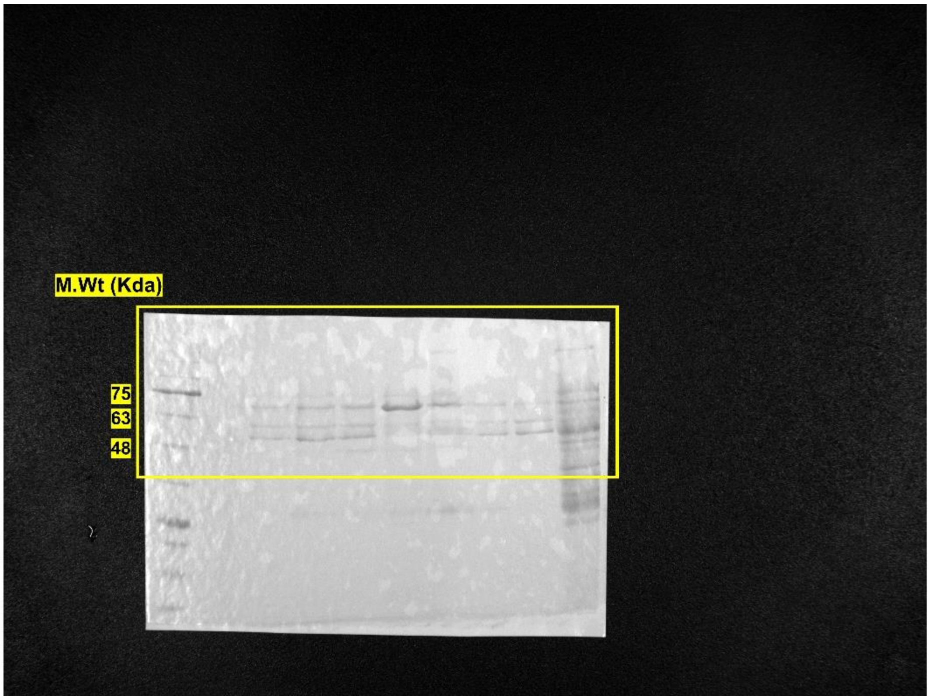

Membrane Cut

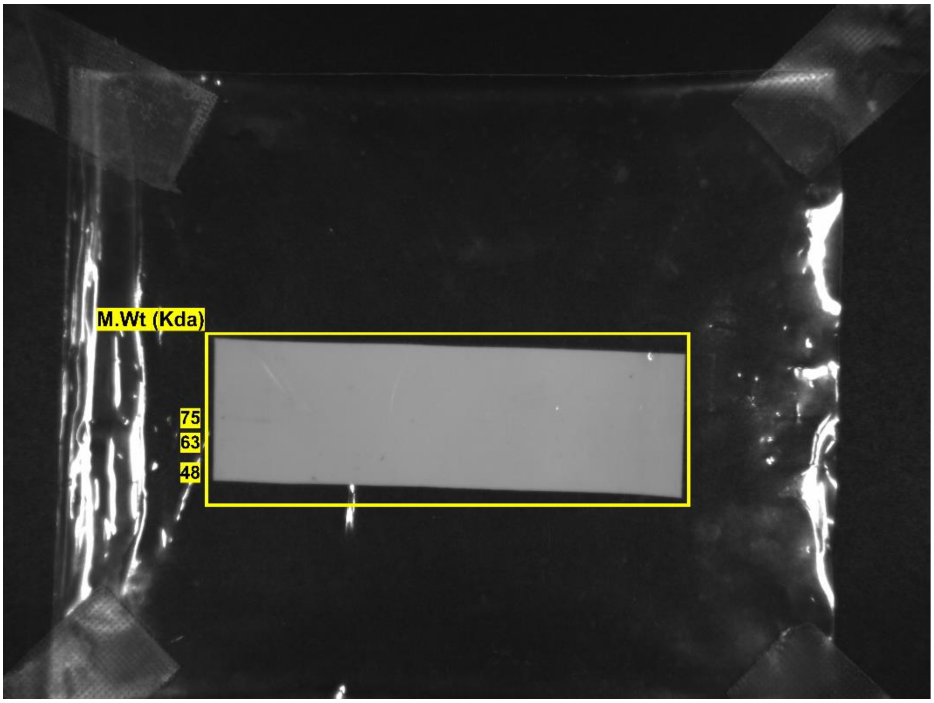

5 min exposure time

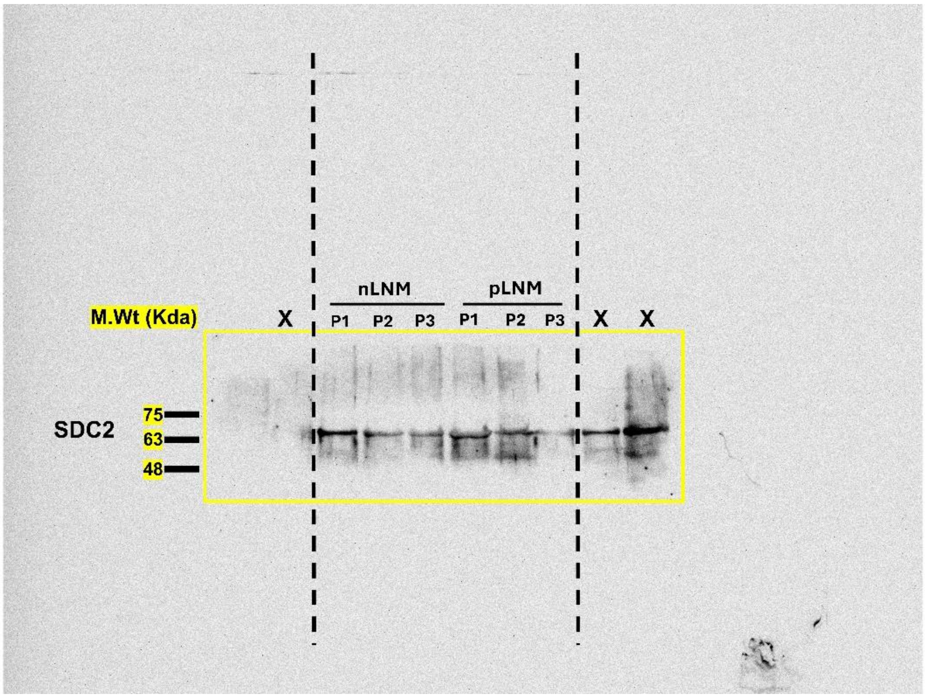

20 min exposure time

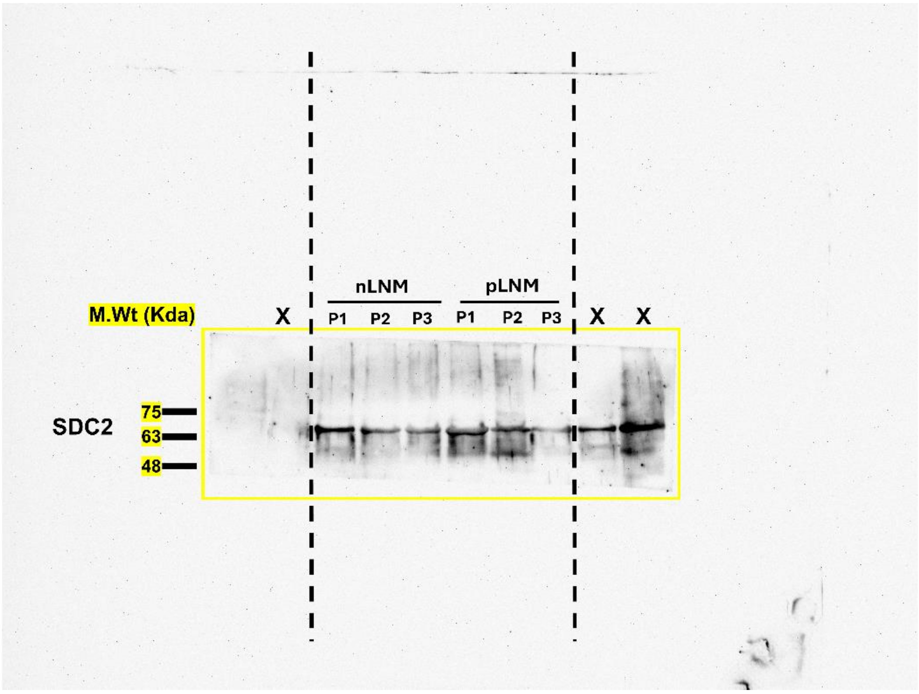

30 min exposure time

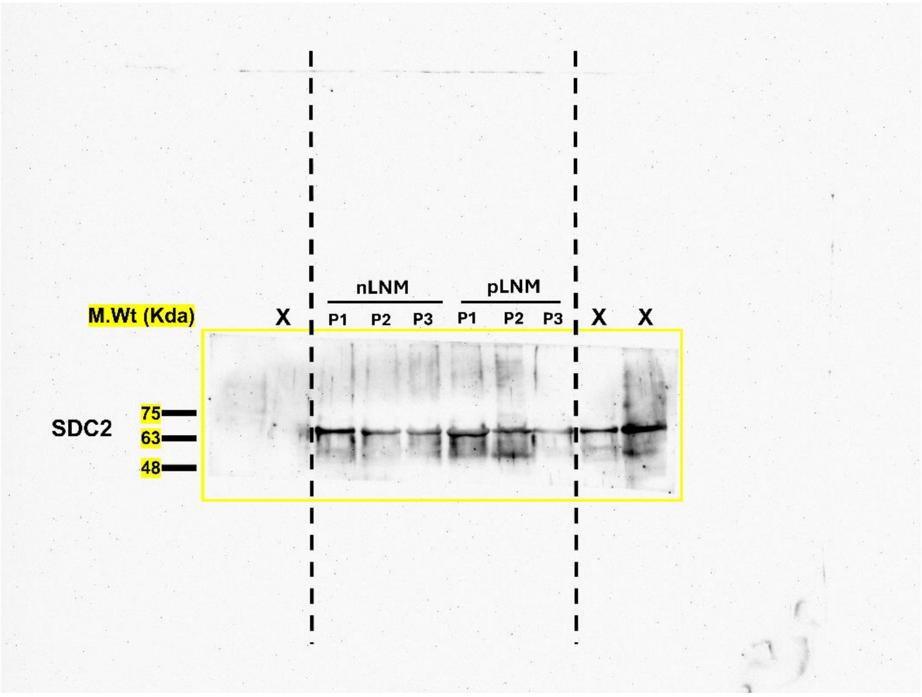

Supplementary Fig. S2b. Raw data for fig. 3B. Western blot analysis of SDC2. Images for Ponceau S staining, membrane cut and blot with varying exposure durations.

Supplementary Fig. S2c. Western blot of CD9

Full membrane Ponceau S stain

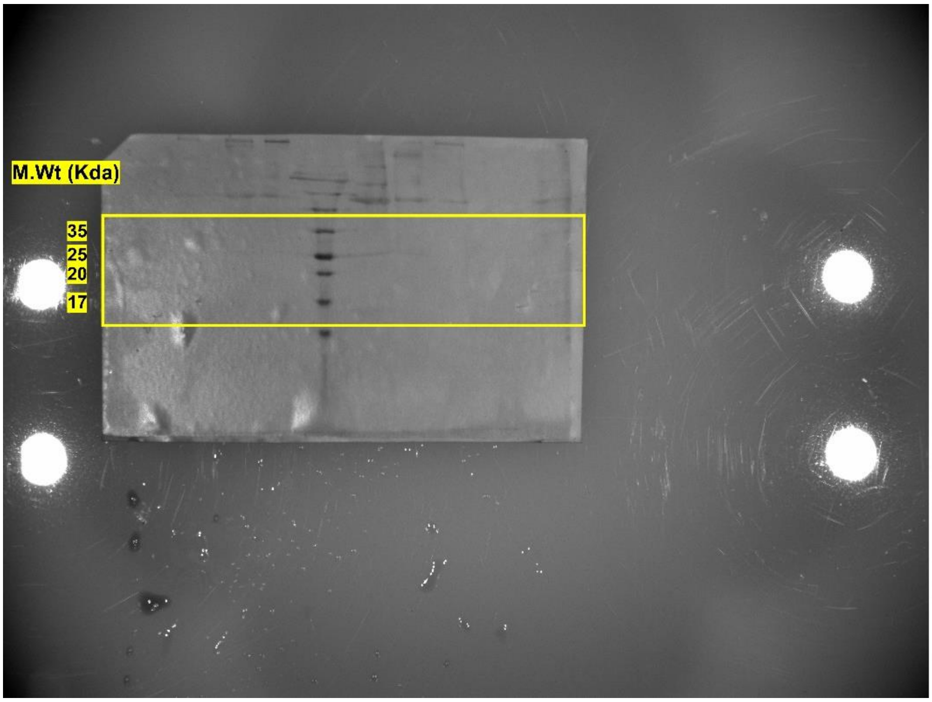

Membrane Cut

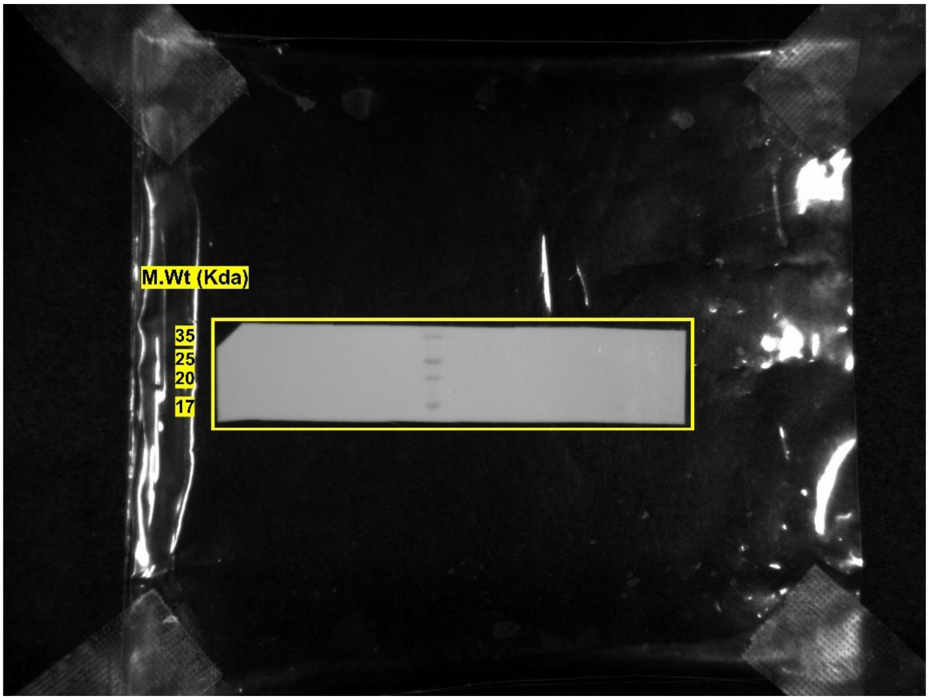

5 min exposure time

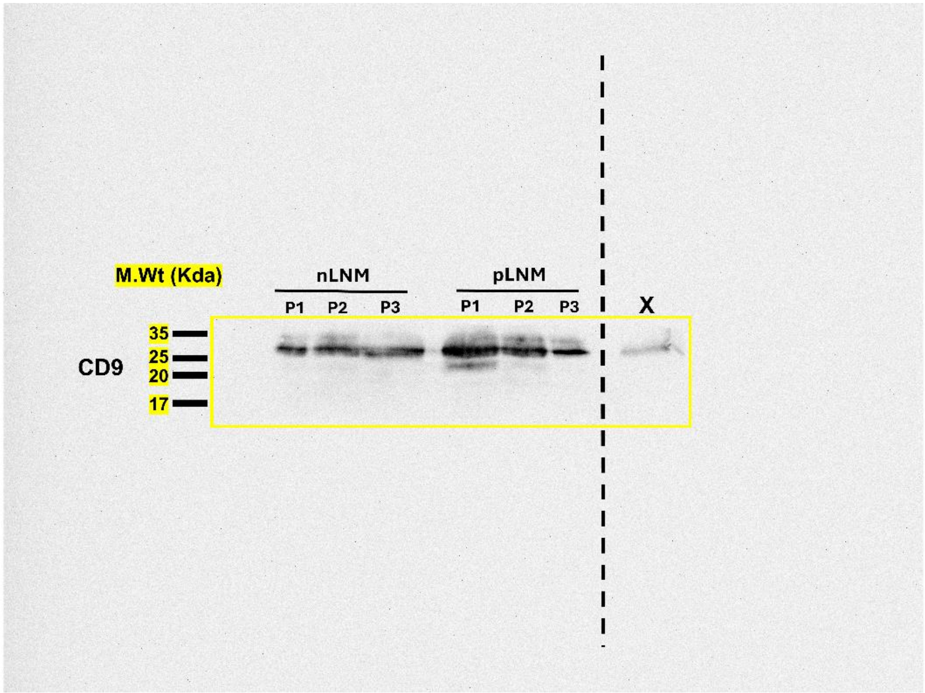

20 min exposure time

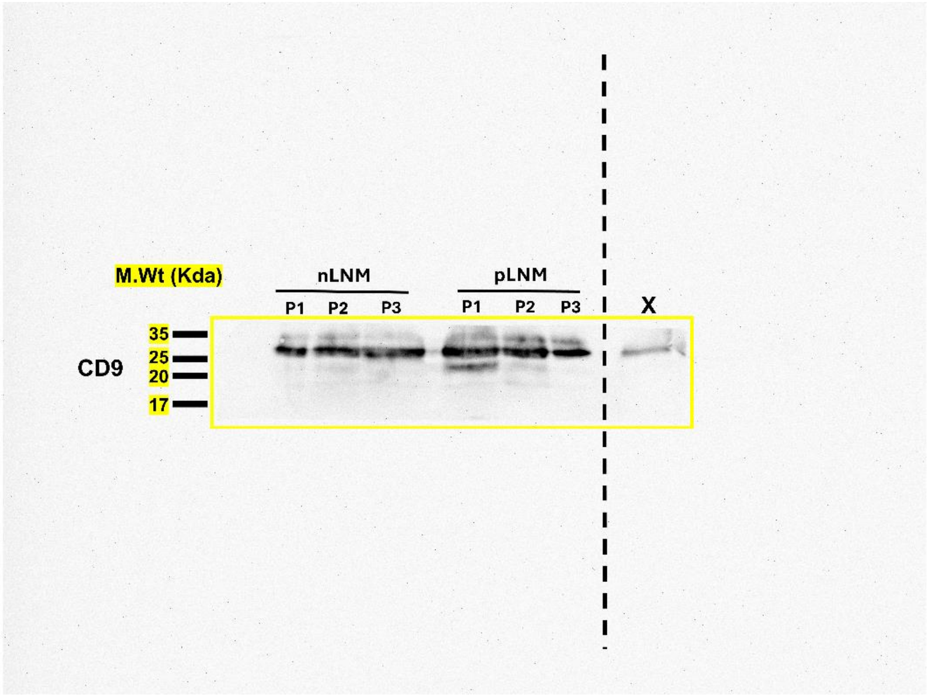

30 min exposure time

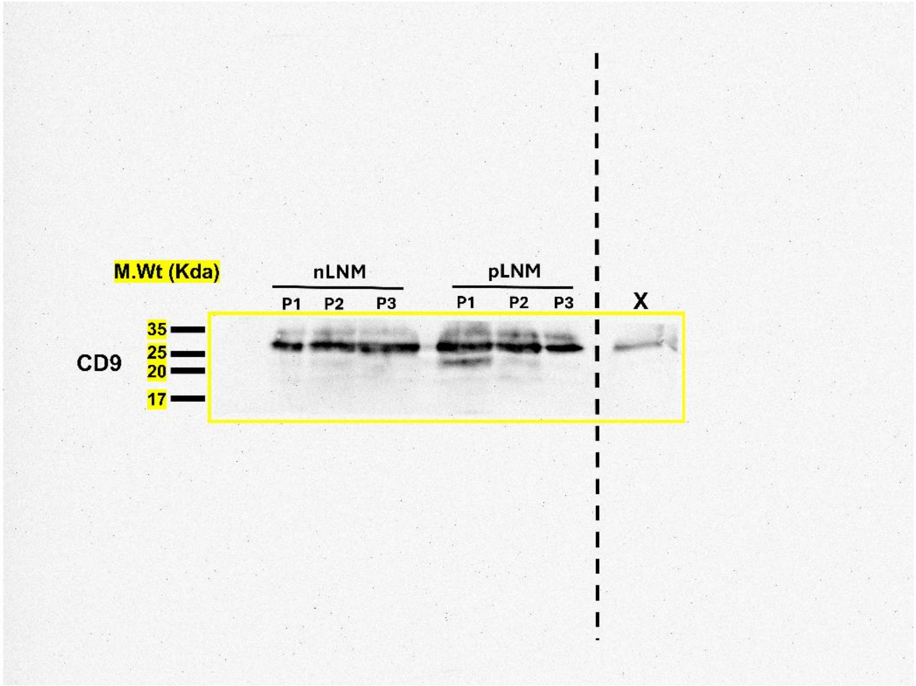

Supplementary Fig. S2c. Raw data for fig. 3B. Western blot analysis of CD9. Images for Ponceau S staining, membrane cut and blot with varying exposure durations.

Supplementary Fig. S2d. Western blot of FN

Replicate 1

Full membrane Ponceau S stain

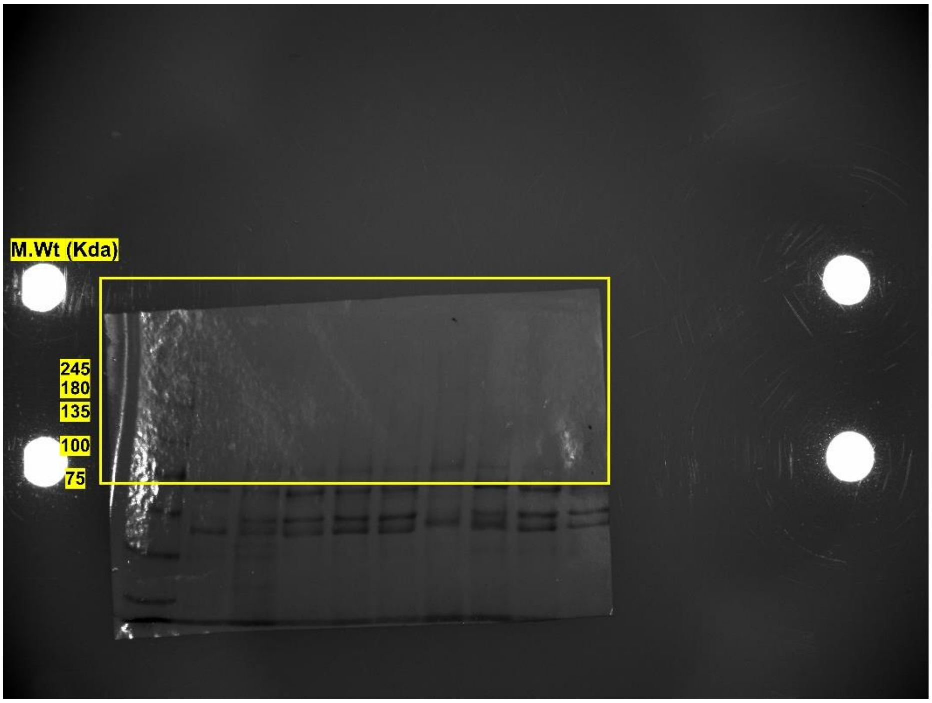

Membrane Cut

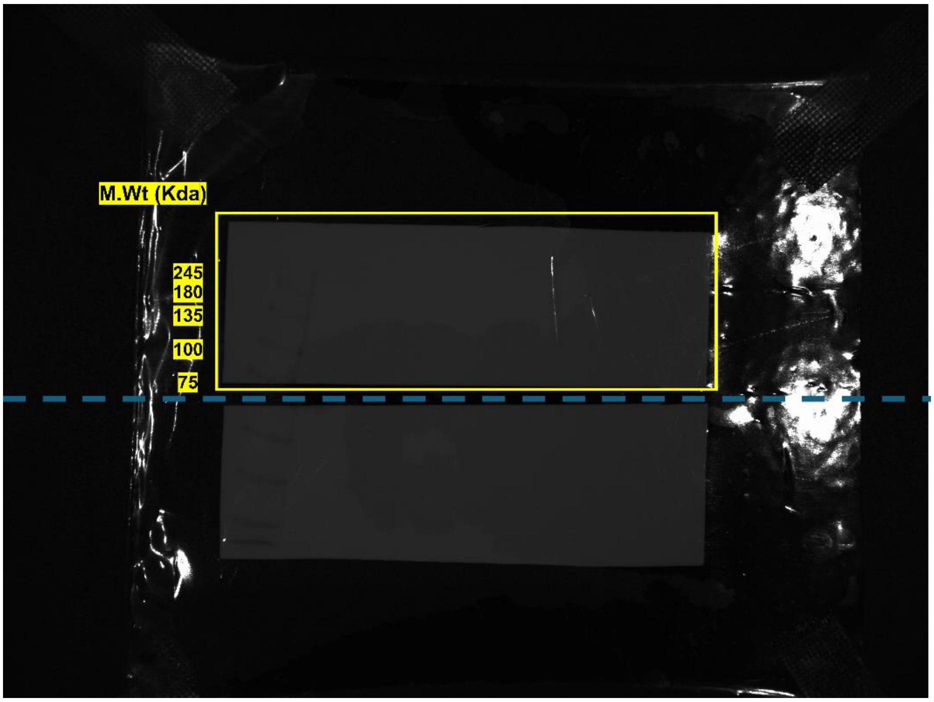

5 min exposure time

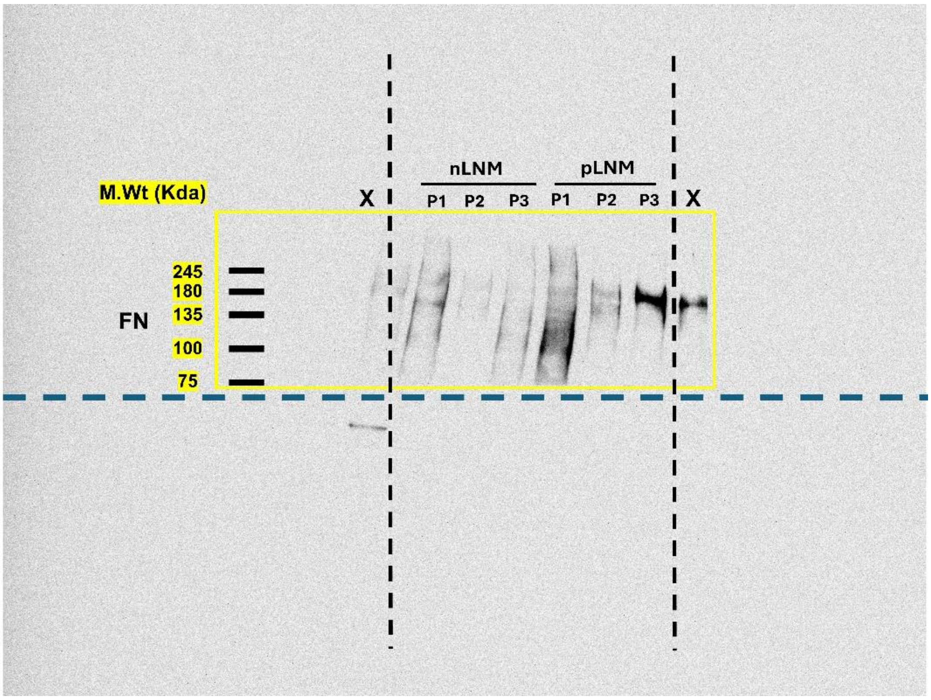

20 min exposure time

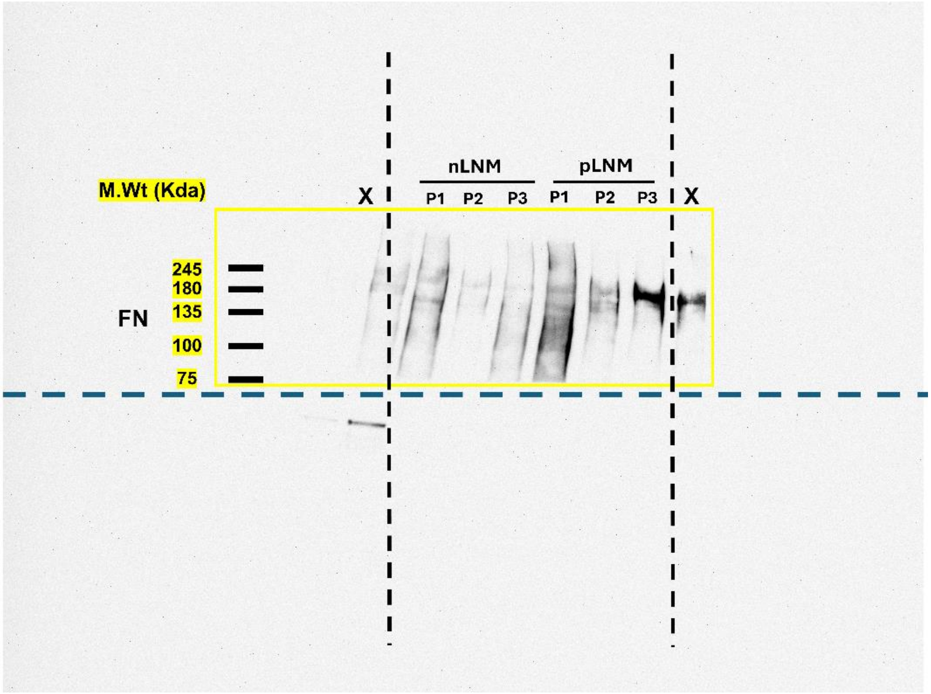

30 min exposure time

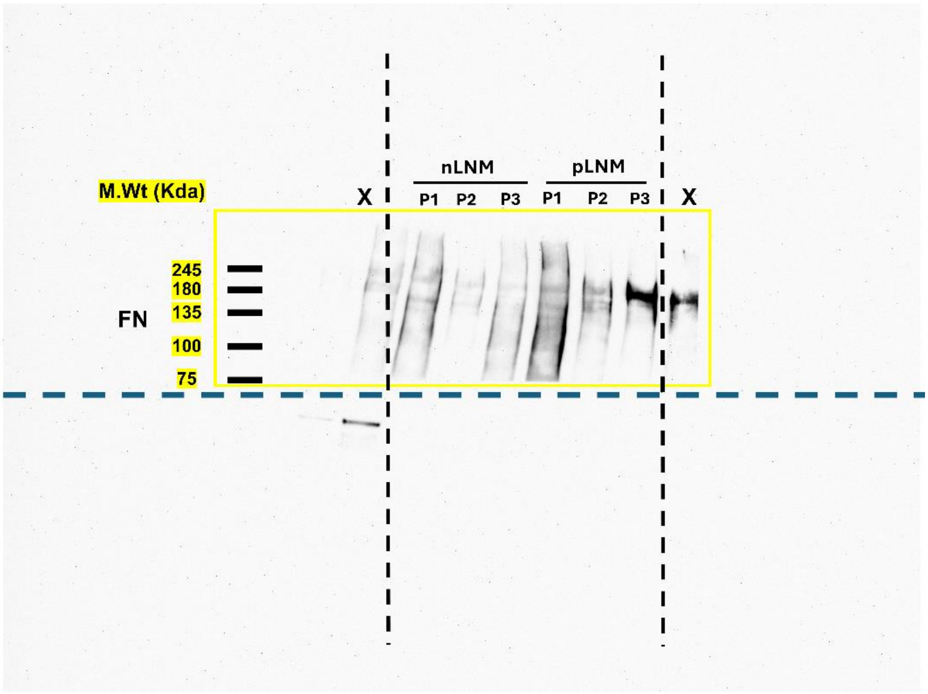

Supplementary Fig. S2d. Raw data for fig. 3E. Western blot analysis of FN. Images for Ponceau S staining, membrane cut and blot with varying exposure durations.

Supplementary Fig. S2d. Western blot of FN

Replicate 2

Full membrane Ponceau S stain

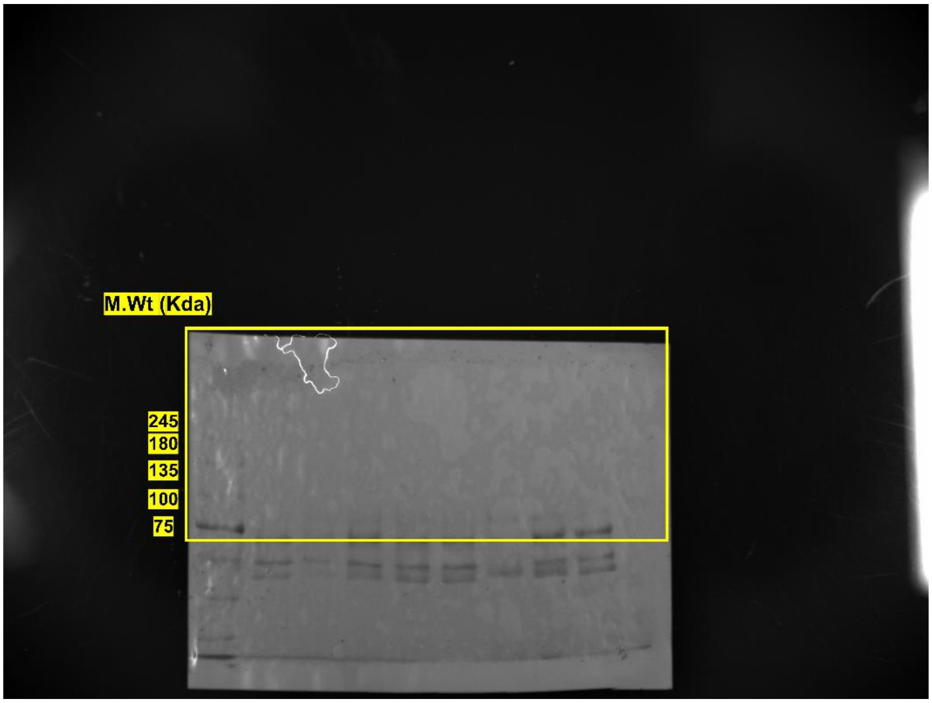

Membrane Cut

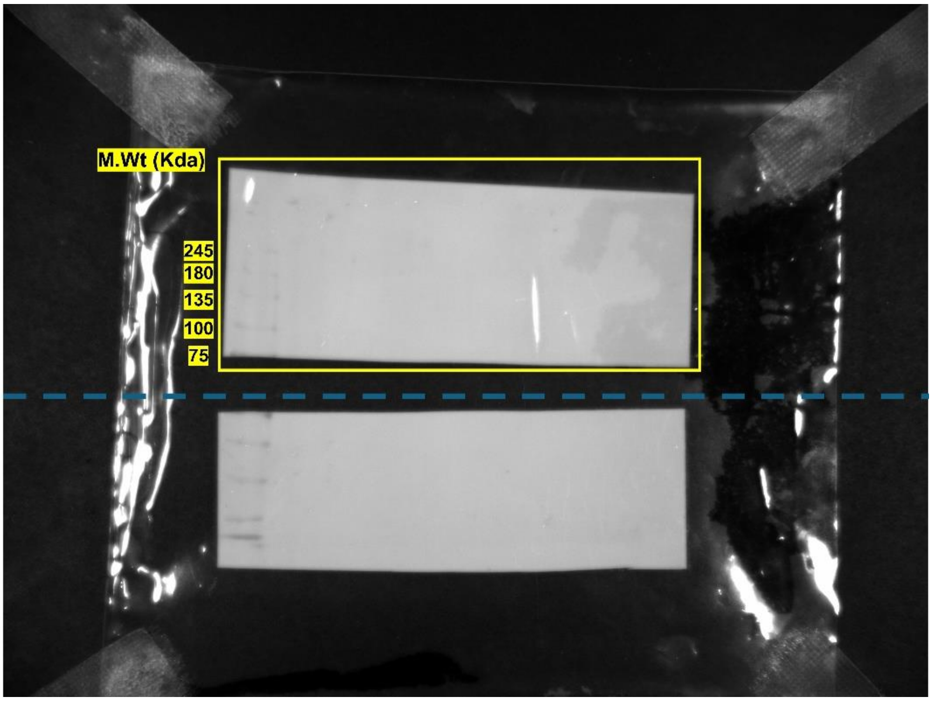

5 min exposure time

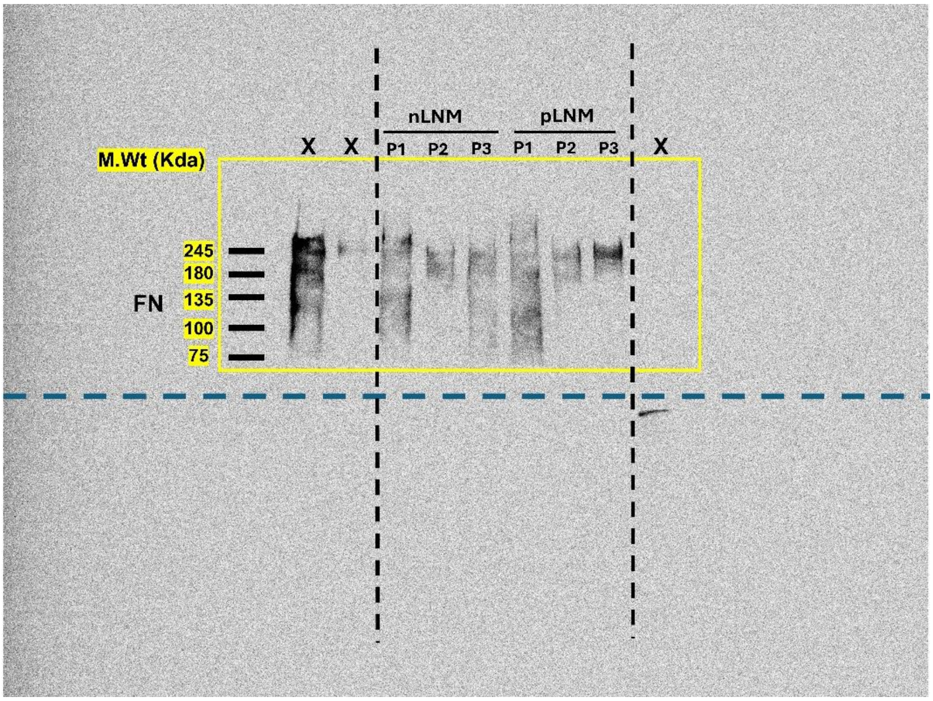

20 min exposure time

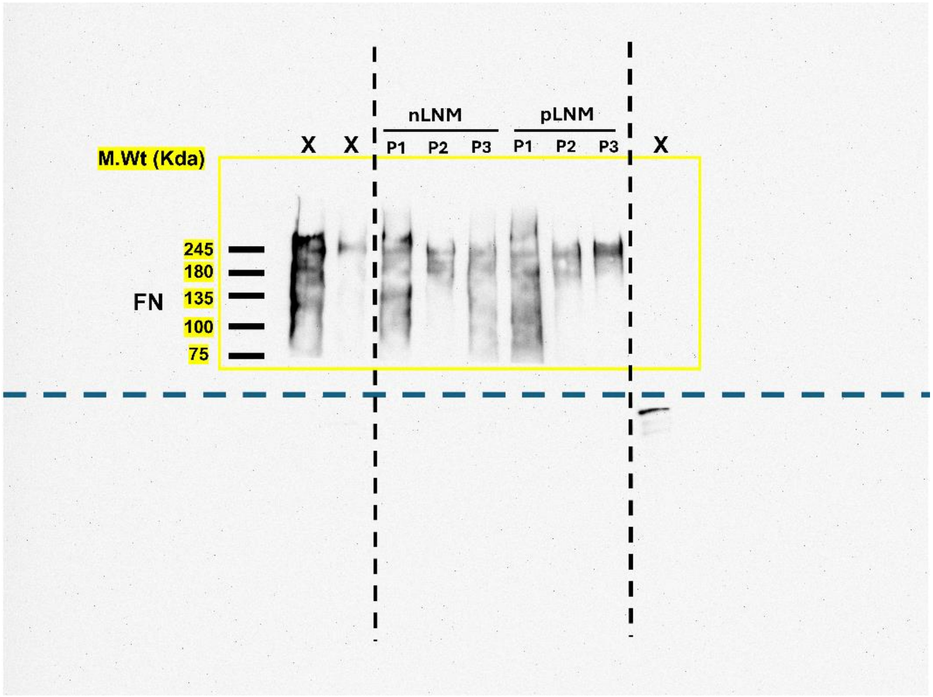

30 min exposure time

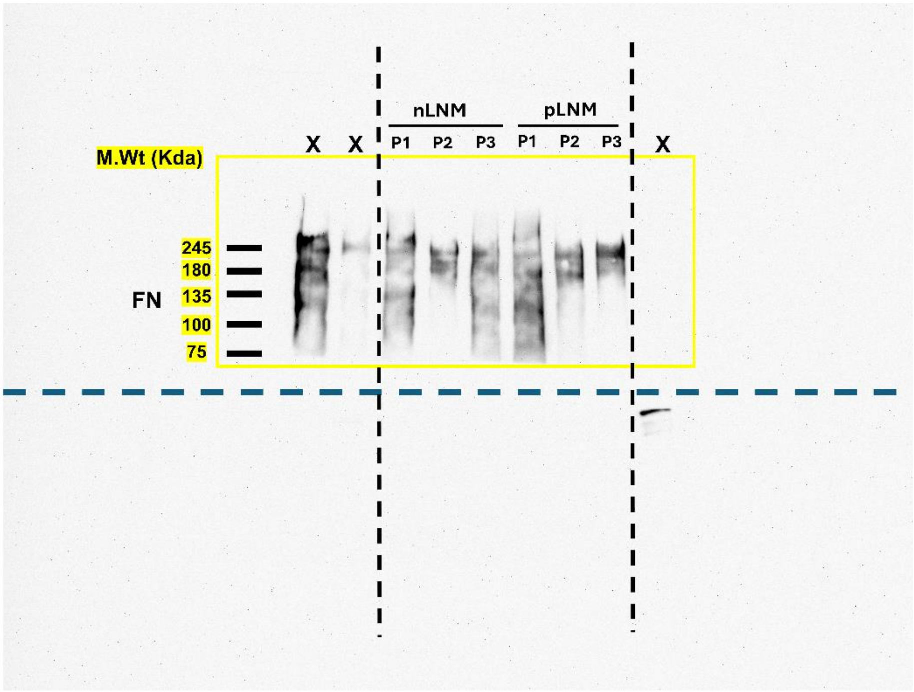

Supplementary Fig. S2d. Raw data for fig. 3E. Western blot analysis of FN. Images for Ponceau S staining, membrane cut and blot with varying exposure durations.

Supplementary Fig. S2e. Western blot of CD9

Full membrane Ponceau S stain

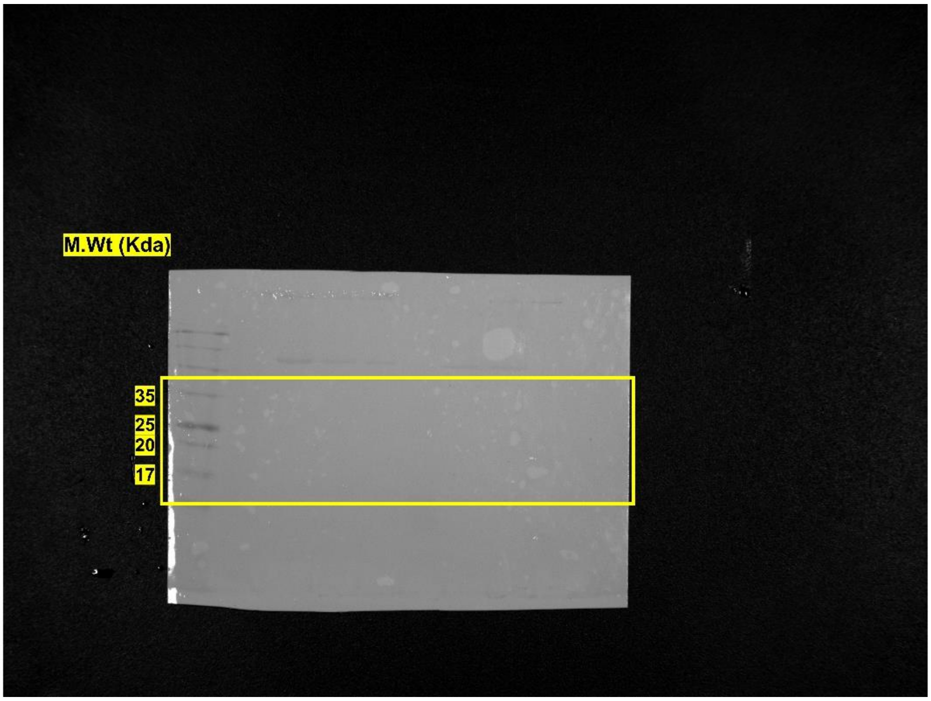

Membrane Cut

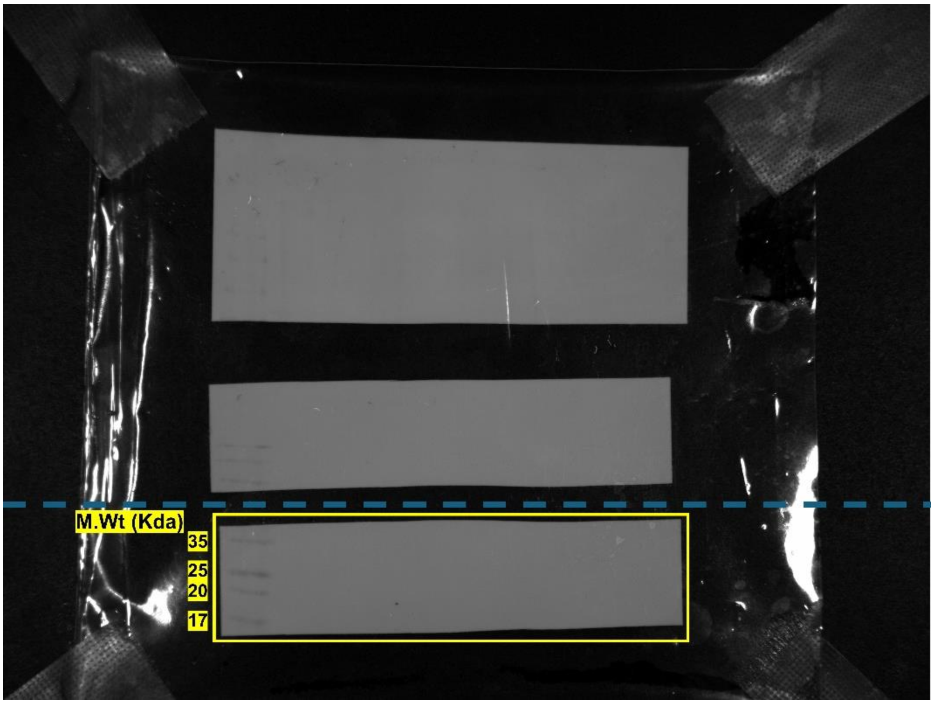

5 min exposure time

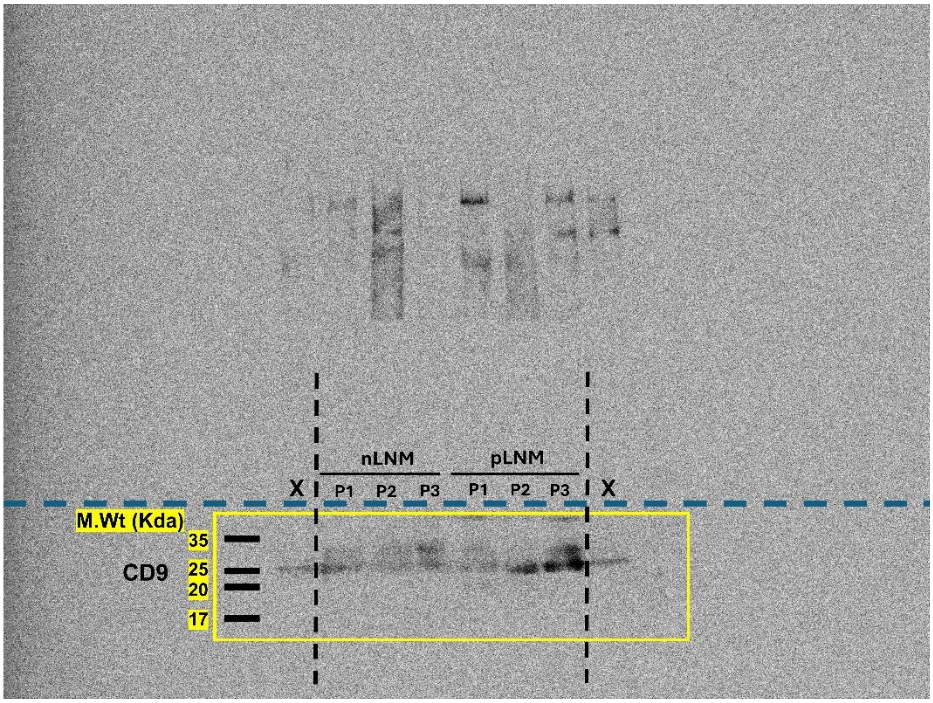

20 min exposure time

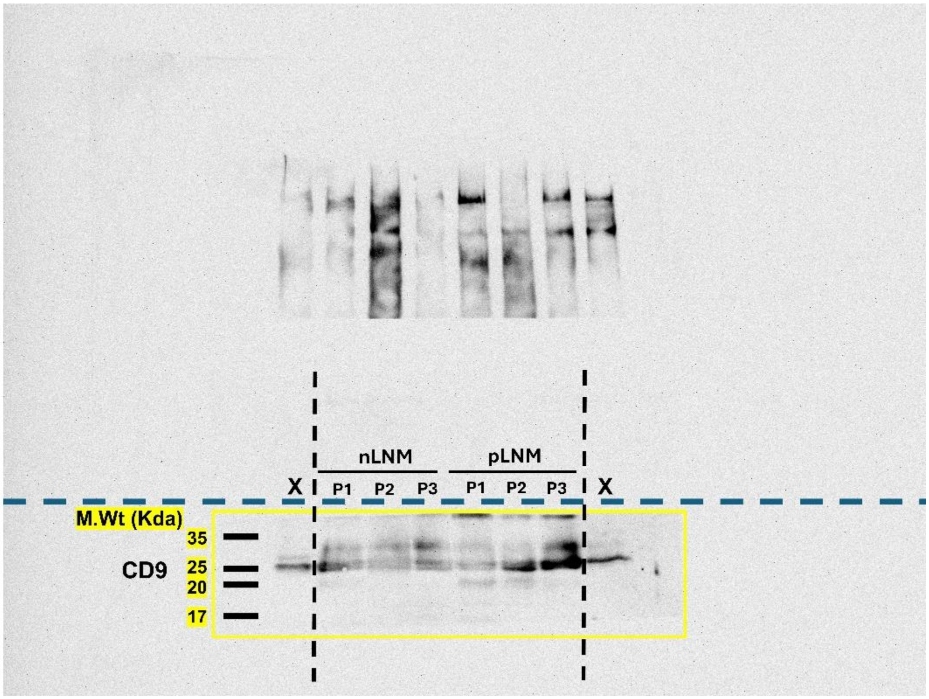

30 min exposure time

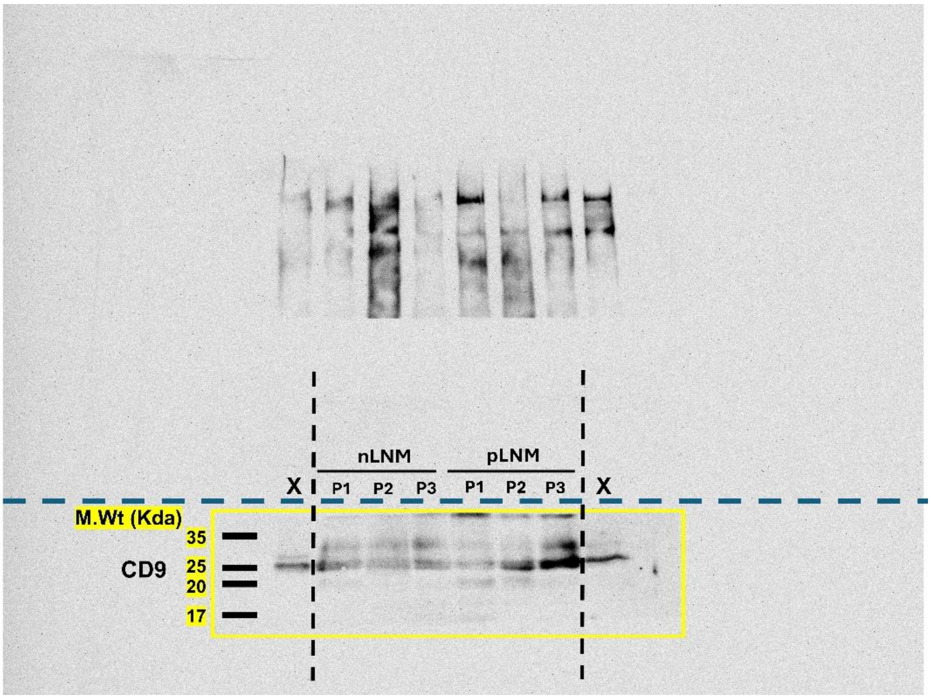

Supplementary Fig. S2e. Raw data for fig. 3E. Western blot analysis of CD9. Images for Ponceau S staining, membrane cut and blot with varying exposure durations.
